# *Edwardsiella piscicida* infection reshapes the intestinal microbiome and metabolome of big-belly seahorses: mechanistic insights of synergistic actions of virulence factors

**DOI:** 10.3389/fimmu.2023.1135588

**Published:** 2023-05-03

**Authors:** Lele Zhang, Fang Wang, Longwu Jia, Hansheng Yan, Longkun Gao, Yanan Tian, Xiaolei Su, Xu Zhang, Chunhui Lv, Zhenhao Ma, Yuanyuan Xue, Qiang Lin, Kai Wang

**Affiliations:** ^1^ School of Agriculture, Ludong University, Yantai, China; ^2^ Research and Development Center of Science, Technology and Industrialization of Seahorses, Ludong University, Yantai, China; ^3^ Department of Pathology, the Affiliated Yantai Yuhuangding Hospital of Qingdao University, Yantai, China; ^4^ Key Laboratory of Tropical Marine Bio-resources and Ecology, South China Sea Institute of Oceanology, Chinese Academy of Sciences, Guangzhou, China

**Keywords:** Edwardsiella piscicida, metagenome, metabolome, virulence factor, big-belly seahorse, pathogenesis

## Abstract

Uncovering the mechanism underlying the pathogenesis of *Edwardsiella piscicida-*induced enteritis is essential for global aquaculture. In the present study, we identified *E. piscicida* as a lethal pathogen of the big-belly seahorse (*Hippocampus abdominalis*) and revealed its pathogenic pattern and characteristics by updating our established bacterial enteritis model and evaluation system. Conjoint analysis of metagenomic and metabolomic data showed that 15 core virulence factors could mutually coordinate the remodeling of intestinal microorganisms and host metabolism and induce enteritis in the big-belly seahorse. Specifically, the Flagella, Type IV pili, and Lap could significantly increase the activities of the representative functional pathways of both flagella assembly and bacterial chemotaxis in the intestinal microbiota (*P* < 0.01) to promote pathogen motility, adherence, and invasion. Legiobactin, IraAB, and Hpt could increase ABC transporter activity (*P* < 0.01) to compete for host nutrition and promote self-replication. Capsule1, HP-NAP, and FarAB could help the pathogen to avoid phagocytosis. Upon entering epithelial cells and phagocytes, Bsa T3SS and Dot/Icm could significantly increase bacterial secretion system activity (*P* < 0.01) to promote the intracellular survival and replication of the pathogen and the subsequent invasion of the neighboring tissues. Finally, LPS3 could significantly increase lipopolysaccharide biosynthesis (*P* < 0.01) to release toxins and kill the host. Throughout the pathogenic process, BopD, PhoP, and BfmRS significantly activated the two-component system (*P* < 0.01) to coordinate with other VFs to promote deep invasion. In addition, the levels of seven key metabolic biomarkers, Taurine, L-Proline, Uridine, L-Glutamate, Glutathione, Xanthosine, and L-Malic acid, significantly decreased (*P* < 0.01), and they can be used for characterizing *E. piscicida* infection. Overall, the present study systematically revealed how a combination of virulence factors mediate *E. piscicida*-induced enteritis in fish for the first time, providing a theoretical reference for preventing and controlling this disease in the aquaculture of seahorses and other fishes.

## 1 Introduction

*Edwardsiella* spp. are the most common and serious gram-negative zoonotic pathogens affecting humans and animals worldwide (1, 2); in particular, they cause more than 90% of fish deaths in aquaculture (3). *Edwardsiella piscicida*, an emerging and important intestinal pathogen in fish (1), causes substantial biomass and economic losses in aquaculture globally (1), seriously threatening the healthy development of fisheries. As *E. piscicida* possesses virulence factors (VFs) similar to many intestinal pathogens, such as Flagella, adherence, invasion, type III and type VI secretion systems (T3SS and T6SS), quorum sensing (QS) regulators, iron uptake, two-component systems (TCS), toxins, and invasion proteins (1–3), it could be used as an attractive model organism to study the role of a single VF or a combination of VFs in pathogenic process (1–3). Unfortunately, current researches have mainly focused on revealing the functional mechanisms of single VFs in *E. piscicida*-induced diseases in mammals (1, 4, 5); the related research in fish is limited. Therefore, exploring the potential mechanisms underlying the mutual interaction of VFs to induce deep infection may not only enrich the knowledge of *E. piscicida*-induced enteritis in fish but also provide theoretical references for development of optimal strategies for the control and prevention of such diseases.

More than one billion microorganisms colonizing the intestinal tract constitute the first barrier against pathogen invasion (6, 7) and are the driving forces of host health (8). As the most dominant and important part of the intestinal microorganisms, bacterial microbiota can act mutually with the immune system and metabolic function of the host (9, 10), as well as the VFs and antibiotic resistance ontologies (AROs) of opportunistic pathogens (11, 12), to maintain intestinal homeostasis (13). However, pathogenic infections can disrupt such homeostasis and cause diseases (11, 14, 15). Multi-omics analyses have been used to reveal pathogenesis mechanisms from the perspective of pathogen-intestinal microbiota-host metabolism in humans and mice (11, 16, 17). Recently, more researches have focused on exploring the relationship among intestinal microbiota, metabolites, and fish diseases by a single-omics approach (14, 18, 19). However, research on multi-omics applications in fish diseases (20) are scant, limiting the characterization of the effects and understanding of mechanisms of intestinal pathogens from a broader molecular level perspective.

As flagship species of the marine ecological environment (21), a drastic decline of the seahorse (*Hippocampus* spp.) population has been observed in the past decade (22). Therefore, establishment of seahorse aquaculture is recommended to provide an alternative source of seahorses (23). Owing to the unique characteristics such as simple intestinal structure and lack of gut-associated lymphatic tissue (24), seahorses easily succumb to bacterial enteritis under crowded farming conditions, causing considerable economic losses (25, 26). To date, over 20 bacterial pathogens, mainly *Vibrio* spp., have been identified, the majority of which can cause enteritis in seahorses (25–28). However, the lack of research on the pathogenesis of bacterial enteritis in seahorses restricts the establishment of effective measures to control and prevent such disease in seahorse aquaculture. In our previous study, we found that *E. tarda* could induce lethal enteritis in farmed lined seahorses (*H*. *erectus*) and established an experimental model to evaluate the pattern and rate of disease progression for the first time (25). Whether such a model is suitable for other seahorses and pathogenic species requires further validation. Our recent research found that *E. piscicida* could also induce lethal enteritis in farmed big-belly seahorses (*H. abdominalis*), posing a major threat to the aquaculture of this species (25). Uncover the pathogenesis of *E. piscicida* induced enteritis in seahorses will be meaningful for the healthy development of the aquaculture industry.

In the present study, we aimed to determine the pathogenic characteristics of *E. piscicida*-induced enteritis in the big-belly seahorses. We enriched and reassessed the current research model and its evaluation system, identified key VFs and AROs, explored their effects on intestinal microbiota and functions through metagenomic analysis, determined the changes in host metabolism, identified key metabolic biomarkers (KMBs) through metabolomic analysis, and illustrated the potential mechanism underlying *E. piscicida*-induced enteritis *via* conjoint analysis. We believe that this study will help enrich our knowledge of *Edwardsiella*-induced enteritis and develop appropriate prevention and control strategies for fish pathogens in aquaculture.

## 2 Materials and methods

### 2.1 Animals and research model construction

Big-belly seahorses were maintained and treated in accordance with the guidelines of Animal Ethics Experimentation approved by the Animal Care and Use Committee of Ludong University (document number: LDU-RB20210308NXY-9). Healthy big-belly seahorses were collected from the Wendeng Seahorse Center of Ludong University, Yantai 264025, Shandong Province, China. Male and female seahorses were maintained in ponds connected to a central circulation system with mechanical and biological filtration, ultraviolet sterilization, and a protein skimmer that continuously aerates water (salinity: 31.5 ± 0.5‰, temperature: 19 ± 0.5°C, pH: 8.2 ± 0.1) at Ludong University for 2 weeks before the experiments. Plastic plants were used as holdfasts. The seahorses were fed three times per day (08:00, 12:00, and 16:00) with frozen *Mysis*, and residual feed and feces were siphoned out 2 h after each feeding session.

To construct the research model, seven groups were set up (20 male and 20 female seahorses per group) to determine the appropriate combination of seahorse size (wet weight, g) and challenge dose of *E. piscicida* (cfu/mL) *via* intraperitoneal (IP) injection (14). In detail, Con represents the combination of 2.5–3.0 g and physiological saline solution, and EPs represent the *E. piscicida* challenge groups with the combinations 3.5–4.0 g and 1 × 10^5^ cfu/mL, 3.5–4.0 g and 1 × 10^7^ cfu/mL, 3.5–4.0 g and 1 × 10^9^ cfu/mL, 4.1–4.5 g and 1 × 10^5^ cfu/mL, and 4.1–4.5 g and 1 × 10^7^ cfu/mL. After the IP injection of *E. piscicida*, the male and female seahorses were separately maintained in tanks (50 × 40 × 40 cm^3^) under the same culture conditions mentioned above, except that the circulating water was turned off. After 24 h, the seahorses were fed normally, feces were siphoned out, and seawater was supplemented to maintain normal water levels. The number of deaths was recorded daily to calculate survival rate, and the seahorses were switched to new tanks every week for 21 days. The appropriate combination for research model construction was determined according to the survival rate and pathological characteristics of the seahorses.

### 2.2 Research model update and sample collection

Using the optimal combination of seahorse size and challenge dose, 200 big-belly seahorses (male: female = 1:1) were equally separated into two groups (Con and EP groups) with 50 males and 50 females per group, and a new research model was constructed. Both growth-related and pathological parameters of the big-belly seahorses listed in the established evaluation system were recorded on days 0, 1, 5, 9, 15, and 21 as previously reported (25). In addition, the respiratory rate (RR) of randomly chosen seahorses per group was recorded daily. After anesthetization with 0.035% MS-222 (Sigma-Aldrich, Saint Louis, Missouri, USA) for 2 min, intestinal samples were collected on days 0, 1, 5, 9, 15, and 21 for further histological and quantitative real-time polymerase chain reaction (q-PCR) analysis. After 21 d, the pathological characteristics of *E. piscicida*-induced enteritis in big-belly seahorses were examined.

The established evaluation system was updated by redefining the scoring range and supplementing the scoring system with new parameters according to our previously established principles (25). Disease activity index (DAI) was determined to reveal the pattern and key pathogenic time points of *E. piscicida*-induced enteritis in big-belly seahorses. To further reveal the pathogenesis of *E. piscicida*-induced enteritis in big-belly seahorses, 60 seahorses (male:female = 1:1) were divided into two groups (Con: 10 males and 10 females; EP: 20 males and 20 females). After repeating the model construction steps, intestinal samples were collected at key pathogenic time points for integrated metagenomics and metabolomics analyses.

### 2.3 Histological observation

The intestine samples randomly collected from the seahorses in the Con and EP groups (n = 3) were fixed in Bouin’s solution. Within 24 h, the tissues were dehydrated in an alcohol-xylene series and embedded in paraffin. The samples from different intestinal segments were cut into 8-μm-thick sections and stained with hematoxylin and eosin (Beyotime, Tianjin, Hebei, China) (25).The sections were then examined under a light microscope (BX53; Olympus, Tokyo, Japan) at 400× magnification to visualize the pathogenic changes in the intestine.

### 2.4 q-PCR analysis and RR calculation

Intestine samples (n = 5) randomly collected from the Con and EP groups on days 0, 1, 5, 9, 15, and 21 were used to extract total RNA using Trizol Reagent (9109, Takara, USA). cDNA was synthesized using the PrimeScript RT Reagent cDNA Amplification Kit with a gDNA Eraser (RR047A; Takara, USA). q-PCR analysis was performed to evaluate gene expression using a SYBR^®^ Premix Ex Taq™ (Takara, Dalian, China) on a Bio-Rad CFX96 Touch machine (Bio-Rad, USA) according to a previously reported method (29). The sequences of the primers used are listed in **Table S1**. The relevant levels of target genes were determined using the 2^−ΔΔCt^ method (30).

Fifteen seahorses from each group were randomly selected, and the frequency of their gill cover movements per minute was recorded to calculate the RR (31).

### 2.5 Metagenomics, metabolomics, and bioinformatics analysis

To eliminate the potential sex-related differences and obtain better matching results of metagenomic and metabolomic sequencing analyses, eight intestinal samples per group, one male and one female seahorses, were randomly collected and thoroughly ground in liquid nitrogen. Half of each sample was used for metagenomic sequencing, and the other half was used for metabolomic sequencing.

To reveal the effects of *E. piscicida* infection on the composition, diversity, structure, function, VF, and ARO of intestinal microbiota, four samples from each group were randomly selected for metagenomic sequencing, and a paired-end sequencing approach was used to obtain raw data (32). Thereafter, data quality control, removal of host genome reads, metagenomic assembly, encoding gene prediction, and non-redundancy gene set construction were performed. Detailed information regarding these steps can be found in the **Supplementary Materials and Methods**. The raw sequencing data of metagenome in this study are available in the Sequence Read Archive database of National Center for Biotechnology Information (NCBI) under accession number PRJNA916642.

To determine the effects of *E. piscicida* infection on host metabolism, liquid chromatography-mass spectrometry (LC-MS) analysis of all eight samples from each group was used for untargeted metabolomic sequencing. Chromatographic separation was performed on a Waters UPLC Acquity I-Class PLUS (Waters Corp., Milford, Connecticut, USA) system with mass spectrometric detection using a Waters UPLC Xevo G2-XS QTOF attachment (Waters Corp.). The samples were analyzed in both positive and negative ion modes. All raw data were collected using MassLynx software (version 4.2, Milford, Connecticut, USA) and entered into Progenesis QI (version 2.4, Munich, Bavaria, Germany) software for further analysis (33).

For the bioinformatic analysis, α-diversity analyses were conducted to evaluate the diversity, evenness, and richness of the intestinal microbiota, especially for the bacteria and their function, VFs and AROs, and metabolites at different levels by using GraphPad Prism (version 8.0, San Diego, California, USA), and β-diversity analyses were conducted to evaluate the structure of the intestinal microbiota, especially for the bacteria and their function, VFs and AROs, and metabolites at different levels by BMK Cloud platform (http://www.biocloud.net/). Linear discriminant analysis effect size (LEfSe) was used to screen biomarkers of intestinal microbiota using the criteria linear discriminant analysis (LDA) > 3 and *P* < 0.05 (34).

Network analysis was employed to visualize the potential connections between bacterial genera and VFs (35). After annotation using the Nr, KEGG, GO, eggNOG, VFDB, and CARD databases (36, 37), correlations between bacterial genera and KEGG functional pathways of intestinal microbiota, as well as VFs and KEGG functional pathways of intestinal microbiota, were assessed using Pearson correlation analysis (https://cloud.majorbio.com/page/tools/). The affiliations of VFs and AROs with bacterial species were determined using data from the non-redundant gene set construction. Potential metabolic biomarkers (PMBs) and KMBs were identified according to previously reported criteria (variable importance in projection [VIP] > 1.0, *P* < 0.05, and VIP > 1.5, *P* < 0.05) (33). The fold change (FC) of KMBs was calculated using the formula E9D/Con.

### 2.6 Statistical analysis

Experimental data are presented as the mean ± standard deviation and analyzed using an independent samples *t*-test in SPSS software (version 23.0, Chicago, Illinois, USA). Structural differences in the intestinal microbiota, function, VF levels, and AROs were determined using the PERMANOVA algorithm (http://www.biocloud.net/). Results with *P* < 0.05 were considered significant and those with *P* < 0.01 were considered highly significant.

## 3 Results

### 3.1 Update of the bacterial enteritis model and determination of the pathogenic characteristics of *E. piscicida* in big-belly seahorses

**Figure 1:**
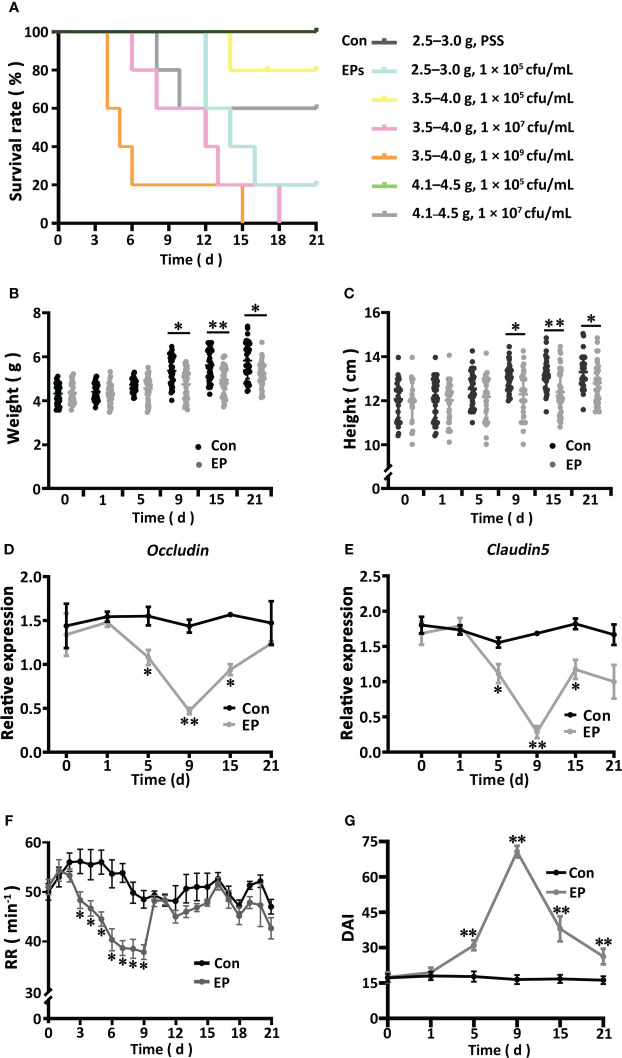
Effects of *Edwardsiella piscicida* infection on the survival **(A)**, body weight **(B)**, body height **(C)**, gene expressions of *Occludin* **(D)** and *Claudin 5* **(E)**, RR **(F)**, and DAI **(G)** of the big-belly seahorse (*H. abdominalis*). In **(A)**, Con represents healthy controls (2.5–3.0 g, PSS), EPs represent *E. piscicida*-challenged groups (2.5–3.0 g, 1 × 10^5^ cfu/mL; 3.5–4.0 g, 1 × 10^5^ cfu/mL; 3.5–4.0 g, 1 × 10^7^ cfu/mL; 3.5–4.0 g, 1 × 10^9^ cfu/mL; 4.1–4.5 g, 1 × 10^5^ cfu/mL; and 4.1–4.5 g, 1 × 10^7^ cfu/mL). In **(B–G)**, Con represents healthy controls (4.1–4.5 g, PSS), and EP represents *E. piscicida*- challenged group (4.1–4.5 g, 1 × 10^5^ cfu/mL). PSS, physiological saline solution; RR, respiratory rate; DAI, disease activity index. *P < 0.05; **P < 0.01.

The survival rate of big-belly seahorses showed a weight- and dose-dependent relationship (**Figure 1A**). The survival rate of the seahorses (4.1–4.5 g) injected with 1 × 10^5^ cfu/mL of *E. piscicida* was the same as that of the Con group (100%), which is suitable for model construction. Whereas, in the other EP groups, the time to death and survival rate appeared to positively and negatively correlate with body weight and challenge concentration, respectively. After the reconstruction of the research model, typical pathological changes associated with bacterial enteritis, such as intestinal epithelial dissolution, focal bleeding, villus atrophy, separation between the lamina propria and submucosa, thickened lamina propria and muscularis mucosae, vascular distorted congestion, and a large amount of inflammatory necrosis in the muscle layer and serosal surface, were observed (**Figure S1**). Compared with those of the Con group, the body weight and length of seahorses in the EP group (4.1–4.5 g and 1 × 10^5^cfu/mL) were reduced, especially on day 9 (*P* < 0.05) (**Figures 1B, C**). The gene expression of intestinal proinflammatory cytokines (*IL-1*;interferon1, *INF1*; tumor necrosis factor-α, *TNF-α*, interleukin-1β, *IL-1β*; IL-1β receptors), anti-inflammatory factors (*IL-10* and *IL-2*), antimicrobial peptides (hepcidin; liver-expressed antimicrobial peptide, *LEAP*; piscidin; lysozyme), and Toll-like receptor 5 (*TLR5*) significantly increased on day 9 and decreased to different extents thereafter (**Figures S2A, B**). At the same time, the gene expression patterns of *ZO-1* were similar to those of the immune genes (**Figure S2C**), whereas the other two tight junction genes claudin 5 and occludin showed the opposite expression patterns (**Figures 1D, E**). The RR of the EP group declined from days 2 to 9, especially from days 3 to 9 (*P* < 0.05), recovered thereafter, and remained at the level of the Con group (**Figure 1F**). As the RR and gene expression of both claudin 5 and occludin varied consistently along with the pathogenic process and other parameters, we updated our previously published evaluation system by adding them to the DAI scoring system (**Table S2**). We found that the DAI of the EP group increased from day 1, became significantly higher than that of the Con group from day 5 (*P* < 0.05), peaked on day 9, and decreased there after until day 21 (**Figure 1G**).

### 3.2 Dysbiosis and biomarkers of intestinal microbiota during *E. piscicida* infection

**Figure 2:**
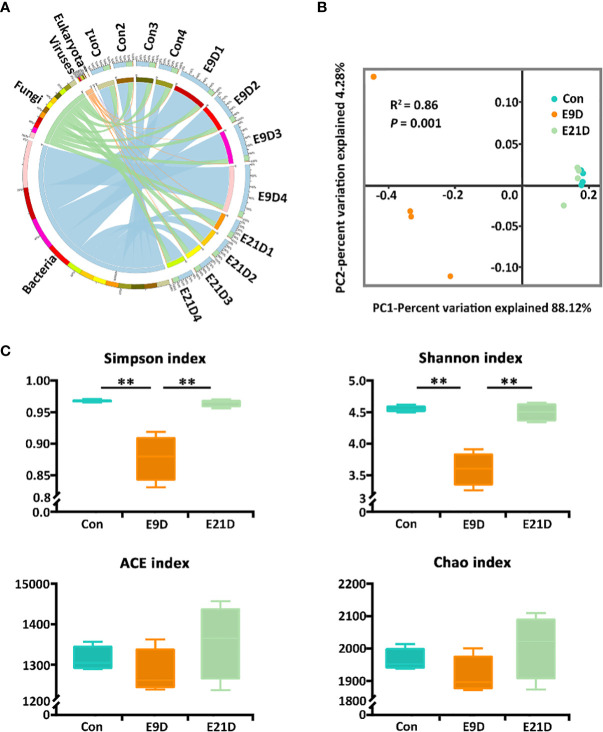
Effects of *Edwardsiella piscicida* infection on kingdom-level composition **(A)**, structure **(B)**, and diversity **(C)** of intestinal microbiota in big-belly seahorses. **(B, C)** represent bacterial intestinal microbiota at the species level. Con represents healthy controls (4.1–4.5 g, PSS); E9D and E21D represent the samples collected on days 9 and 21 of *E. piscicida*- challenged group (4.1–4.5 g, 1 × 10^5^ cfu/mL), respectively (similarly hereinafter). **P < 0.01.

A total of 256,229 genes were identified *via* sequencing, belonging to 6 kingdoms, 95 phyla, 119 classes, 250 orders, 552 families, 1329 genera, and 3171 species (**Figure 2A**; **Table S4**). The number of sequences could reflect the structure and diversity of the microbial community because the corresponding rarefaction curves had already reached the saturation plateau (**Figure S3A**). The α-diversity and β-diversity of the intestinal microorganisms of the E9D group were significantly different from those of the Con and E21D groups at the species level (*P* < 0.01), whereas the difference between the Con and E21D groups was not significant (*P* > 0.05) (**Figures S3B, C**).

The intestinal microbiota composition at the kingdom level was numerically dominated by bacteria in each group (**Figure 2A**). As the strains used for stress were bacterial pathogens, all subsequent analyses were based on bacterial levels. There were 2066 shared bacterial species in the Con, E9D, and E21D groups, with 231, 227, and 380 endemic species, respectively (**Figure S3D**). As shown in **Figure 2B**, the β-diversity of the intestinal microbiota in the E9D group was significantly different from that of the Con and E21D groups at the species level (*P* < 0.01) but not between the Con and E21D groups (*P* > 0.05). The Shannon and Simpson indices of α-diversity in the E9D group were significantly lower than those of the Con and E21D groups (*P* < 0.01), but the ACE and Chao richness indices were not significantly different among the three groups (*P* > 0.05) (**Figure 2C**). This result indicates that pathogen infection altered the structure, and significantly reduced the evenness and diversity of intestinal microbiota in seahorses.

**Figure 3:**
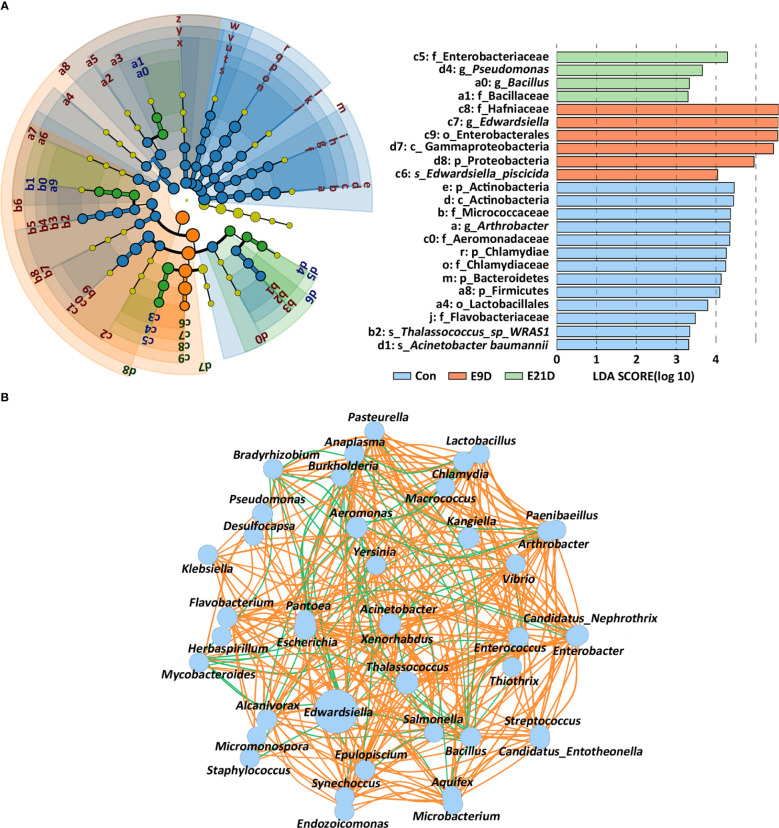
Effects of *Edwardsiella piscicida* infection on the expression of biomarkers **(A)** and interaction structure **(B)** of the intestinal microbiota in seahorses. The node size displays the abundance of the species. Intestinal microbiota biomarker screening criteria: *P* < 0.05, LDA > 3. The color of the line reflects the direction in the B-plot (*P* < 0.05, |r| > 0.7): orange, positive; green, negative.

The composition of the intestinal microbiota at different levels changed after *E. piscicida* infection (**Figure S4**). The LEfSe analysis revealed significant differences in the phylogenetic distribution and biomarkers of the microbiota among the groups (*P* < 0.05) (**Figure 3A**). Notably, *Edwardsiella* and *Edwardsiella piscicida* accounted for 70.10% and 32.74% of the relative abundance in the identified bacteria, respectively (**Table S5**). *Edwardsiella* abundance was significantly positively correlated (*P* < 0.05, |r| > 0.7) with *Chlamydia*, *Enterobacter*, *Yersinia*, *Pantoea*, *Salmonella*, *Xenorhabdus*, *Klebsiella*, and *Arthrobacter* but negatively correlated (*P* < 0.05, |r| > 0.7) with *Lactobacillus*, *Microbacterium*, *Enterococcus*, *Acinetobacter*, *Mycobacteroides*, *Flavobacterium*, and *Thalassococcus* (**Figure 3B**).

### 3.3 Relationship between biomarkers and functions of the intestinal microbiota during *E. piscicida* infection

**Figure 4:**
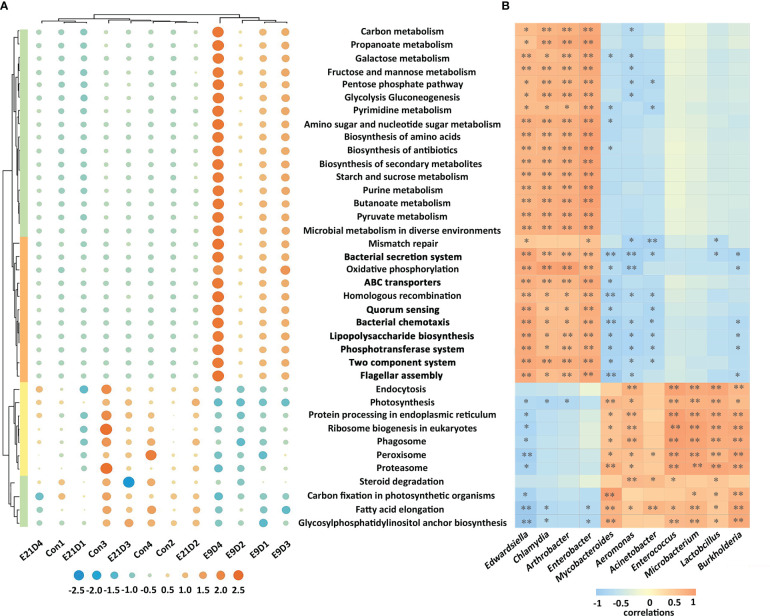
Effects of *Edwardsiella piscicida* infection on the functions **(A)** and its correlation with biomarkers **(B)** of intestinal microbiota in seahorses. In **(A)** the significance level of the KEGG functional activity difference of intestinal microbiota not in bold typeface was *P* < 0.05, whereas that in bold typeface was *P* < 0.01. The color column represents the KEGG level 1 functional classification (green: metabolism; orange: cellular process and environmental information processing; yellow: genetic information processing). The blue circles represent a decrease in relative abundance, whereas the orange circles represent an increase in abundance. In B, values > 0 represent a positive correlation, whereas values < 0 represent a negative correlation. Darker colors also indicate a stronger correlation. *P < 0.05; **P < 0.01.

The structure and activity of the functional pathways of the intestinal microbiota in the E9D group were significantly different from those of the Con and E21D groups at all three annotated KEGG, GO, and eggNOG levels (*P* < 0.01) (**Figure 4A**; **Figure S5**). As shown in **Figure 4**, compared with that of the Con group, the relative abundance of *Edwardsiella*, *Chlamydia*, *Enterobacter*, and *Arthrobacter* (*P* < 0.05) and the activities of 28 positively correlated functional pathways (*P* < 0.01, |r| > 0.7) were significantly increased (*P* < 0.01), whereas the relative abundance of *Lactobacillus*, *Enterococcus*, *Microbacterium*, *Acinetobacter*, *Mycobacteroides*, *Aeromonas*, and *Burkholderia* and the activities of 11 positively correlated functional pathways (*P* < 0.05, |r| > 0.7) were significantly decreased (*P* < 0.05) in the E9D group. Notably, the activities of eight functional pathways, including bacterial chemotaxis, flagellar assembly, lipopolysaccharide biosynthesis, phosphotransferase system, bacterial secretion system, QS, ABC transporters, and TCS, increased significantly (*P* < 0.01) in the E9D group (**Figure 4**), indicating the crucial role of these pathways during *E. piscicida* infection.

### 3.4 Key VFs (KVFs) and their relationship with intestinal microbiota function

Metagenomic analysis identified 10 categories, 181 VFs, and 478 virulence genes (**Table S6**; **Figure S6A**). The structure of VFs in the E9D group was significantly different from that of the Con and E21D groups (*P* < 0.01) (**Figure S6B**), which was similar to the results of the intestinal microbiota. Significant differences in α-diversity were found between the Con and E9D groups (*P* < 0.01) (**Figure S6C**; **Table S6**), consistent with the results of the intestinal microbiota and their functions.

**Figure 5:**
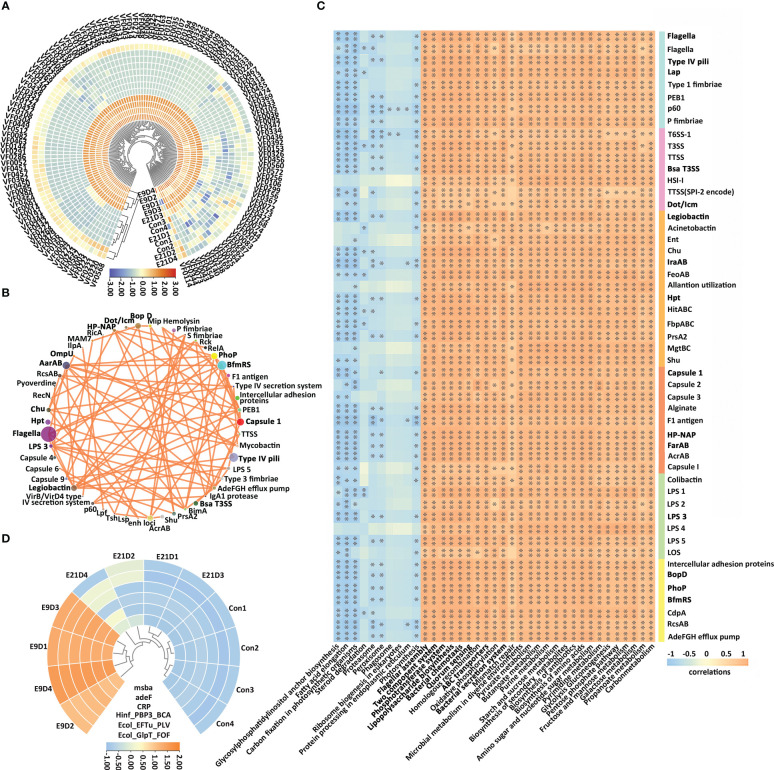
Effects of *Edwardsiella piscicida* infection on the composition and abundance of virulence factors (VFs) **(A)**, the correlation between VFs **(B)**, the correlation between VFs of relative abundance (TOP 50) and microbiota function **(C)**, and abundance of antibiotic resistance ontologies (*P* < 0.05) **(D)** of the intestinal microbiota. The color in **(A)** represents the relative VF content (blue, decrease; orange, increase). The 15 VFs in bold typeface in **(B)** represent their core role in regulating other VFs and the functions of intestinal microbiota. The orange lines among VFs indicate significant positive correlations (*P* < 0.05, |r| > 0.7). Colored columns on the right of **(C)** represent the classification of VFs (blue, motility, adherence, and invasion; purple, effector delivery system; orange, iron uptake system; red, immune modulation; green, toxin; yellow, regulation). Values > 0 represent a positive correlation, whereas values < 0 represent a negative correlation. Darker colors also indicate a stronger correlation. *P < 0.05; **P < 0.01.

Compared with that of the Con group, the relative abundance of 128 VFs in the E9D group significantly increased (*P* < 0.05), including 126 VFs that were extremely significantly increased (*P* < 0.01) (**Figure 5A**; **Table S6**). *Edwardsiella* could express 123 VFs covering all 10 categories (**Table S6**) and all six pathogenic processes. Significant positive correlations were observed among 50 VFs (*P* < 0.05, |r| > 0.7) (**Figure 5B**). The relative abundance of 15 of them, Flagella, Type IV pili, Lap, Bsa T3SS, Dot/Icm, FarAB, Capsule1, Hp-NAP, Legiobactin, IraAB, Hpt, LPS3, PhoP, BfmR, and BopD, was in the top 50 and complex nodes (*P* < 0.05) (**Figure 5B**; **Table S6**), indicating their pivotal role in regulating other VFs. As shown in **Figure 5C**, the top 50 most abundant VFs were significantly positively (*P* < 0.01, |r| > 0.7) and negatively (*P* < 0.05, |r| > 0.7) correlated with 28 significantly increased and 11 significantly decreased (*P* < 0.05) intestinal microbiota functions (**Figure 4**), respectively, indicating the importance of these VFs in regulating intestinal microbiota function. In addition, 48 AROs were annotated (**Figure S7A**; **Table S7**). The structure of AROs in the E9D group was significantly different from that in the Con and E21D groups (*P* < 0.05) (**Figure S7B**), which is similar to the results of the intestinal microbiota and VFs. The Chao and Shannon indices of α-diversity in the E9D group were significantly higher than those in the control group (*P* < 0.01) (**Figure S7C**). Six AROs with significantly increased abundance (*P* < 0.05) in the E9D group were identified (**Figure 5D**), five of which were expressed by *Edwardsiella*, adeF, msbA, Ecol_EFTu_PLV, Ecol_GlpT_FOF, and Hinf_PBP3_BCA (**Table S7**).

### 3.5 Variations in host metabolites and function of KMBs

**Figure 6:**
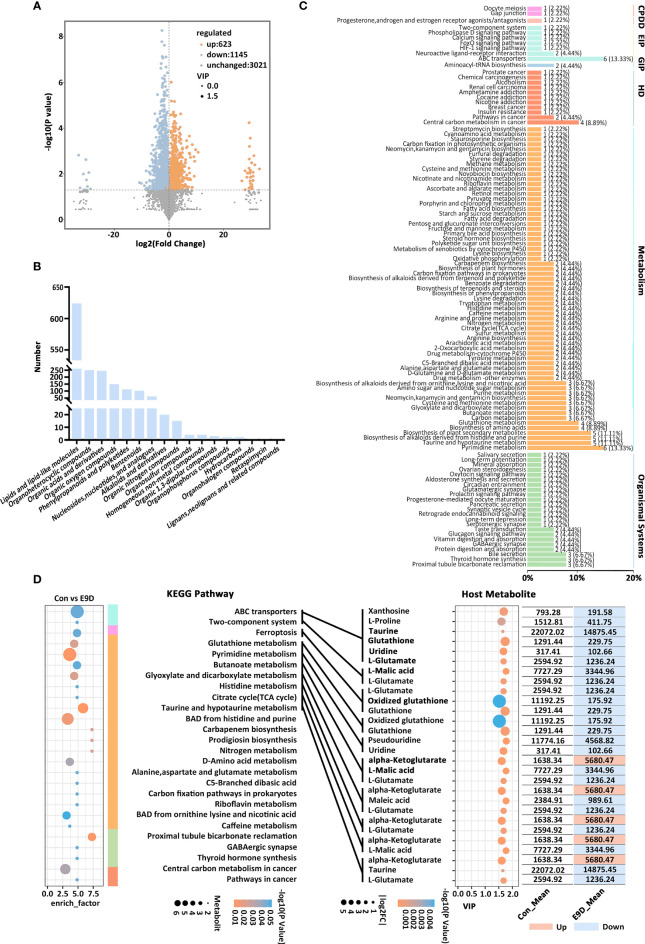
Effects of *Edwardsiella piscicida* infection on the composition **(A)**, classification **(B)**, KEGG functional classification of seahorse intestinal potential metabolite biomarkers (PMBs) **(C)**, and the content of key metabolite biomarkers (KMBs) and their KEGG functions **(D)**. CP, cellular processes; EIP, environmental information processing; GIP, genetic information processing; VIP, variable importance in projection. BAD, biosynthesis of alkaloids derived. The color column represents the KEGG level 1 functional classification in **(D)** (green, environmental information processing; blue, cellular processes; light blue, metabolism; orange, organismal systems; yellow, human diseases). The size the circle on the left side represents the levels of metabolites, whereas the size of the circle on the right side represents the |log2FC|.

Metabolomic data revealed that the structure of metabolites of the E9D groups was different from that of the Con group (**Figures S8A–C**). A total of 17 categories, 4789 metabolites, and 1768 PMBs (VIP > 1.0 and *P* < 0.05; 623 upregulated and 1145 downregulated) were identified (**Figures 6A, B**, **S8D**; **Table S8**). At the same time, 491 KMBs (VIP > 1.5 and *P* < 0.05; 189 upregulated and 302 downregulated) were identified (**Figure S8E**), indicating that *E. piscicida* infection significantly affected metabolic processes in big-belly seahorses.

Forty-eight out of the 491 KMBs (**Table S9**) were enriched in six major functional categories and 110 functional pathways (**Figure 6C**). The activities of 27 functional pathways from 5 major functional categories were also significantly different (*P* < 0.05) (**Figure 6D** left panel). Eleven KMBs (abundance TOP 15 and |log2FC | > 0.55) were identified and enriched in 10 significantly different functional pathways (*P* < 0.05) (**Figure 6D**). Compared with those of the Con group, KMBs associated with histidine metabolism, citrate cycle (TCA cycle), and taurine and hypotaurine metabolism pathways were mostly upregulated. In contrast, KMBs associated with ABC transporters, TCS, ferroptosis, glutathione metabolism, and pyrimidine metabolism pathways were all downregulated, whereas KMBs related to glyoxylate and dicarboxylate metabolism and butanoate metabolism pathway were mostly downregulated in the E9D group (**Figure 6D**). Notably, eight KMBs, alpha-Ketoglutarate, Taurine, Glutathione, Uridine, L-Glutamate, L-Malic acid, Glutathione, and Oxidized glutathione, were involved in regulating multiple functional pathways (**Figure 6D** right panel), suggesting essential roles during *E. piscicida* infection.

### 3.6 Molecular pathogenesis of *E. piscicida*-induced enteritis

**Figure 7:**
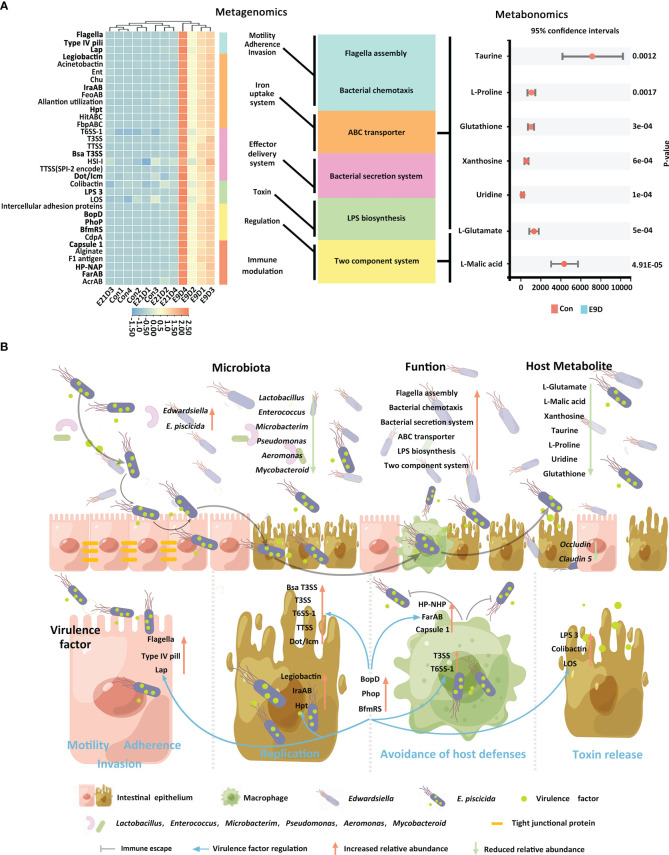
Correlation among core functions of the intestinal microbiota, key virulence factors, and key metabolites of the host **(A)**. Significant positive correlations *P* < 0.01, |r| > 0.8. Schematic diagram of pathogenic mechanisms **(B)** of *Edwardsiella piscicida* infection. *Edwardsiella piscicida* infection could be divided into six stages: motility, adherence, invasion, replication, avoidance of host defenses, and toxin release. The KVFs Flagella, Type IV pili, and Lap allow pathogen approach, attachment, and binding to host epithelial cells, and invasion and internalization into the villus epithelial cells. After entering epithelial cells, legiobactin, IraAB, and Hpt help compete for more nutrients to improve pathogen survival and replication in a nutrient-deficient environment. Meanwhile, T3SS, T6SS-1, TTSS, HSI-I, VirB/VirD, and Dot/Icm promote pathogen replication and invasion to the adjacent epithelial cells and other tissues. Upon invasion into deeper tissues, pathogens could encounter phagocytes. HP-NAP, Capsule1, and FarAB could help pathogens avoid phagocytosis, whereas T3SS and T6SS-1 could help them survive and replicate within phagocytes. After release, they could destroy the intestinal barrier (characterized by the downregulations of *Occludin* and *Claudin 5*). After successful colonization, LPS3, Colibactin, and LOS could help release toxins, induce severe inflammatory responses, and finally lead to host death. Core VFs BopD, Phop, and BfmRS could coordinate with other VFs in responding to environmental changes to accelerate deep tissue infection. When a large number of *E. piscicida* is released into the intestinal lumen, it can induce enteritis by increasing the relative abundance of related pathogens and virulence factors and changing the composition structure, function, and host metabolism of the intestinal microbiota.

Centered on the eight closely related key functions of the intestinal microbiota, we found 34 KVFs significantly positively correlated with six of them (*P* < 0.01, |r| > 0.8), including15 were core VFs (CVFs) and the other 19 of the top 50 VFs in abundance. As shown in **Figure 7A**, 34 KVFs among the top 50 VFs with relatively complex nodes correlated with six key intestinal microbiota functions and seven KMBs after *E. piscicida* infection. For instance, in the E9D group, the relative abundance of VFs associated with motility, adhesion, and invasion of pathogens significantly and simultaneously increased with the functional activity of flagella assembly and bacterial chemotaxis (*P* < 0.01). The relative abundance of the effector delivery system-related VFs significantly and simultaneously increased with the functional activity of the bacterial secretion system (*P* < 0.01). The relative abundance of the iron uptake system-related VFs increased with the functional activity of ABC transporters of the intestinal microbiota (*P* < 0.01), whereas the levels of L-Proline, Taurine, Glutathione, Uridine, Xanthosine, and L-Glutamate significantly decreased (*P* < 0.05). Similarly, the relative abundance of toxin-related VFs significantly and simultaneously increased with the functional activity of LPS biosynthesis. The relative abundance of the regulation-related VFs significantly and simultaneously increased with the functional activity of TCS of the intestinal microbiota (*P* < 0.01), whereas the levels of KMBs L-Malic acid and L-Glutamate significantly decreased (*P* < 0.05) (**Figures 4D** left panel; **5C**; **7A**). In addition, seven of the eight KMBs with central roles were enriched and significantly negatively correlated with two key intestinal microbiota functions, TCS and ABC transporters (*P* < 0.05). The content of L-Malic acid and L-Glutamate related to TCS significantly decreased after *E. piscicida* infection (*P* < 0.05), as did the levels of L-Proline, Taurine, Glutathione, Uridine, Xanthosine, and L-Glutamate in relation to ABC transporters (*P* < 0.05) (**Figure 7A**).

## 4 Discussion

In this study, we found that *E. piscicida* infection inhibited growth and induced typical pathological features of enteritis in big-belly seahorses, similar to our previous findings on *E. tarda-*induced enteritis in lined seahorses (25). The RR of the big-belly seahorses was significantly reduced in the deep infection stage, indicating inhibition of basal metabolic activity after *E. piscicida* infection (31). Significant downregulation of occludin and claudin 5 expression on day 9 of *E. piscicida* infection might have led to alterations in intestinal permeability and increased bacterial paracellular flux (38). As a component of tight junctions, the expression of *ZO-1* significantly decreases after pathogen infection in fish (39). Interestingly, *ZO-1* expression in the present study was significantly upregulated in the deep infection stage, consistent with previous reports in mice (40), suggesting that *ZO-1* is dispensable for barrier function and can act as a regulator of tight junctions to promote the repair of damaged mucosa (41). In addition, the DAI results suggest that the updated evaluation system can effectively reflect the pattern and intensity of disease progression. Different from *E. tarda*-induced enteritis in lined seahorses (25), more severe pathological features and sooner peaked DAI were found in *E. piscicida*-induced enteritis in the big-belly seahorses under similar challenge conditions. This finding may be attributed to the higher toxicity of *E. piscicida* or a relatively smaller body proportion of the trunk area of big-belly seahorses with pathogen infection than lined seahorses.

Systematic studies exploring relevant omics datasets will enable scientist to describe the complexity and characteristics of interactions in the host-pathogen network, and identify new targets or biomarkers for pathogenic infections (20, 42). In the present study, *E. piscicida* infection could alter the composition, structure, and abundance and significantly reduce the diversity of the intestinal microbiota of big-belly seahorses. These results are similar to previously reported pathogenic infections in lined seahorses, yellow seahorses (*H. kuda*), largemouth bronze gudgeon (*Coreius guichenoti*), and pearl gentian grouper (*Epinephelus lanceolatus ♂ × E. fuscoguttatus ♀*) (14, 16, 26, 43). Lipopolysaccharide (LPS) is a key VF and an important component of the outer membrane of gram-negative pathogens (44). Bacterial chemotaxis plays a crucial role at all stages of infection (45). Flagellar assembly is essential for bacterial pathogens to reach the optimal infection sites, promote biofilm formation, and adhere to host surfaces or cells (46). The bacterial secretion system can interfere with host immunity and disrupt or mimic host cellular processes, and help *Edwardsiella* avoid phagocytosis and replicate within phagocytes and epithelial cells of the intestine (2, 47), inducing severe systemic infections and killing the host (48). The phosphotransferase system is involved in stimulating biofilm formation, aggregating motility, and inducing pathogen colonization (49). ABC transporters, as nutritional VFs, help bacterial pathogens absorb nutrients, such as iron, vitamins, and metabolites, and grow and survive in nutrient-limited or harsh environments (50, 51). TCS is the primary control switch in signal transduction, physiology, cell-cell communication, adaptation to changing environments, and pathogenesis of bacterial pathogens (52). QS is a major regulator of natural competence, motility, and virulence, and controls the switch between the replicative and transmissive/virulent phases of *Legionella pneumophila* (53). In the present study, *E. piscicida* infection significantly increased the relative abundance of the opportunistic pathogens (*Edwardsiella*, *Chlamydia*, *Enterobacter*, and *Arthrobacter*) and the activities of their positively correlated 28 functional pathways, but decreased the abundance of the probiotic microbiota (*Enterococcus*, *Microbacterium*, *Lactobacillus*, and *Burkholderia*) and opportunistic pathogens (*Aeromonas*, *Acinetobacter*, and *Mycobacteroides*). This suggests that *E. piscicida* infection may enhance the competition for nutrients against autochonous microbiota and certain pathogenic bacteria by increasing the activities of functional pathways such as chemotaxis and QS, induce dysbiosis of the intestinal microbiota, and finally cause enteritis.

VFs are key features for the selective advantage of potentially pathogenic bacteria over common members of healthy gut microbiota (54). In the present study, we identified 123 VFs that were significantly increased in abundance after *E. piscicida* infection for the first time, of which 15 CVFs may play a key role in pathogenesis. Referring on previously reported VFs of other bacterial pathogens, *E. piscicida* may rely on Flagella, Type IV pili, and Lap for adhesion and infection (55, 56); Bsa T3SS and Dot/Icm for invasion, intracellular replication, and immune escape (57, 58); Legiobactin, IraAB, and Hpt for survival and growth (46, 59, 60); HP-NAP, Capsule1, and FarAB for evasion of host immune defenses and killing (61–63); and LPS3 for poisoning and killing the host (63). In addition, PhoP, BfmRs, and BopD may help *E. piscicida* sense environmental changes and coordinate the expression of other VFs (64–67). Finally, *E. piscicida* infection may reshape the intestinal microbiota and its functions and induce enteritis through the synergistic regulation and crosstalk between the 15 key VFs and other VFs. In addition, the five AROs of *Edwardsiella* may resist or evade the bactericidal effects of tetracycline, fluoroquinolone, elfamycin, fosfomycin, nitroimidazole, and beta-lactam antibiotics through mechanisms such as active efflux of antibiotics, transferring to other resistant bacteria, and biofilm formation (68, 69). More attention should be paid to these characteristics when designing prevention and control strategies against *Edwardsiella* infections.

The metabolite composition of organisms can provide substantial evidence for identifying biomarkers in pathological processes and analyzing pathogenic mechanisms (70). In this study, *E. piscicida* infection resulted in dysfunctions in metabolism, environmental information processing, and cellular processes, consistent with the results of *E. tarda* infection in tilapia (*Oreochromis mossambicus*) (71). The ABC transporters are associated with the absorption of nutrients, vitamins, and metabolites (51). TCS regulates magnesium ion levels, pH, and antimicrobial peptide-associated signaling (72), converting external signals into gene expression in a dose-dependent manner (73). Pyrimidine metabolism is essential for DNA and RNA synthesis (74). In this study, the functional activities of ABC transporters, TCS, and pyrimidine metabolism were significantly downregulated, suggesting the inhibition of host nutrient transport systems, environmental changes, immune-related signal transduction systems, and translation mechanisms during *E. piscicida* infection. Ferroptosis and glutathione metabolism are essential for pathogen defense in the host, and the downregulation of their activities could disrupt intestinal homeostasis (75, 76) and promote the invasion of *E. piscicida*. In addition, the TCA cycle, histidine metabolism, and taurine and hypotaurine metabolism were altered after *E. piscicida* infection, indicating disturbance in host energy metabolism (77) and immune defense mechanisms (78, 79). Collectively, these results suggest that *E. piscicida* infection affects energy metabolism and disrupts the defense mechanisms of big-belly seahorses by inhibiting nutrient transport, signal transduction, and translation to promote virulence regulation, replication, and transmission.

Pathogen infection can generally be divided into six stages: motility, adherence, invasion, replication, avoidance of host defenses, and toxin release (2), during which significant associations among VFs, intestinal microbiota, and metabolism are found (54, 80, 81). To the best of our knowledge, this is the first study to provide evidence that 34 KVFs are closely related to six key intestinal microbiota functions and seven host KMBs during *E. piscicida* infection in big-belly seahorses. Significant upregulation of KVFs associated with motility, adherence, and invasion may allow *E. piscicida* to adhere, bind to host intestinal epithelial cells, and invade or be internalized into host tissues and cells by increasing the activities of bacterial chemotaxis and flagellar assembly of the intestinal microbiota (45, 55). With the aggravation of local infection, the upregulation of KVFs associated with the effector delivery system and iron uptake system could help pathogens acquire nutrients for growth, survival, and proliferation under nutrient-limited and harsh host environments by enhancing the functional activity of pathogenic ABC transporters (51, 60). Meanwhile, local infection increases the activity of the bacterial secretion system, allowing the pathogen to survive and replicate in epithelial cells and spread to the adjacent epithelial cells and other tissues (1, 3). During deep invasion, *E. piscicida* may avoid phagocytosis by upregulating KVFs associated with immune regulation (82) or survive and replicate within phagocytes with the help of KVFs of the effector delivery system (1, 83). Through these two mechanisms, *E. piscicida* may successfully avoid attack by the host immune system and disrupt the host mucosal and microbial barriers. Moreover, significant increases of both the abundance of toxin KVFs and LPS biosynthesis results in the production of large amounts of LPS, leading to host tissue necrosis (2, 63). During the invasion process, the upregulation of KVFs may activate the TCS pathway, and thereby help signal transmission and activate appropriate VFs according to environmental conditions by regulating their transcriptional activity (1, 47, 52). The synergy of these processes increases the abundance and accelerates the deep infection of *E. piscicida*.

Taurine, L-Proline, and Uridine have antibacterial activity and can improve host resistance against pathogen invasion (6, 84, 85); therefore, they can be used as KMBs to characterize host resistance to infection by *Edwardsiella* spp. and other pathogens. L-Glutamate is essential for the host to maintain intestinal health and improve growth performance and survival (86). Glutathione has an antioxidant function and can be used to characterize the energy production of host mitochondria (87). Xanthosine and L-Malic acid can provide carbon and energy sources for the growth of pathogens (88, 89). In the present study, all seven KMBs were involved in multiple metabolic pathways and were significantly downregulated after infection, suggesting that *E. piscicida* can use metabolites from big-belly seahorses to promote its replication and infection while inhibiting the energy production and disease resistance of the host. These KMBs may act as typical metabolic features to characterize *E. piscicida* infection. In addition, KMBs related to ABC transporters and TCS pathway-related genes were significantly altered during deep infection, suggesting a key role for these two functions in that specific infection stage. Applying the analysis pattern employed in this study to investigate other infection stages may help identify more KMBs and better understand the underlying mechanisms of the pathogenic process. In summary, we drew a schematic figure to help interpret the mechanisms underlying *E. piscicida* infection-induced enteritis in big-belly seahorses (**Figure 7B**).

## 5 Conclusions

In this study, we updated the current research model and determined the pathogenic patterns and pathological characteristics of bacterial enteritis induced by *E. piscicida* infection in big-belly seahorses; elucidated the role of KVFs in regulating the diversity, structure, and function of intestinal microbiota; and determined the changes and KMBs of host intestinal metabolites for characterizing *E. piscicida* infection. Our results shed light on the pathogenetic mechanisms underlying *E. piscicida*-induced bacterial enteritis in big-belly seahorses. This may enrich the knowledge and provide theoretical references for preventing and controlling related diseases.

## Data availability statement

The datasets presented in this study can be found in online repositories. The names of the repository/repositories and accession number(s) can be found below: https://www.ebi.ac.uk/ena/browser/view/PRJNA916642.

## Ethics statement

The animal study was reviewed and approved by the Animal Care and Use Committee of Ludong University (document number: LDU-RB20210308NXY-9).

## Author contributions

LZ and FW, conceptualization, methodology, investigation, formal analysis, data curation, visualization, resources, writing - original draft, writing- review and editing. LJ, investigation, formal analysis, data curation, visualization, and resources. HY and LG, visualization, data curation, and investigation. YT and XS, investigation and formal analysis. XZ, investigation. CL, sample collection and investigation. ZM, visualization. YX, resources. QL, writing-review and editing and conceptualization. KW, supervision, validation, funding acquisition, writing-review & editing, and conceptualization. All authors contributed to the preparation of article and approved the submitted version.
