# Corrigendum: *Edwardsiella piscicida* infection reshapes the intestinal microbiome and metabolome of big-belly seahorses: mechanistic insights of synergistic actions of virulence factors

**DOI:** 10.3389/fimmu.2023.1222662

**Published:** 2023-06-15

**Authors:** Lele Zhang, Fang Wang, Longwu Jia, Hansheng Yan, Longkun Gao, Yanan Tian, Xiaolei Su, Xu Zhang, Chunhui Lv, Zhenhao Ma, Yuanyuan Xue, Qiang Lin, Kai Wang

**Affiliations:** ^1^ School of Agriculture, Ludong University, Yantai, China; ^2^ Research and Development Center of Science, Technology and Industrialization of Seahorses, Ludong University, Yantai, China; ^3^ Department of Pathology, the Affiliated Yantai Yuhuangding Hospital of Qingdao University, Yantai, China; ^4^ Key Laboratory of Tropical Marine Bio-resources and Ecology, South China Sea Institute of Oceanology, Chinese Academy of Sciences, Guangzhou, China

**Keywords:** Edwardsiella piscicida, metagenome, metabolome, virulence factor, big-belly seahorse, pathogenesis

In the published article, there was an error in **Figure 2A** as published. E9 1-4 and E21 1-4 should be changed to E9D 1-4 and E21D 1-4, respectively. The corrected Figure 2 and its caption appear below.

**Figure 2:**
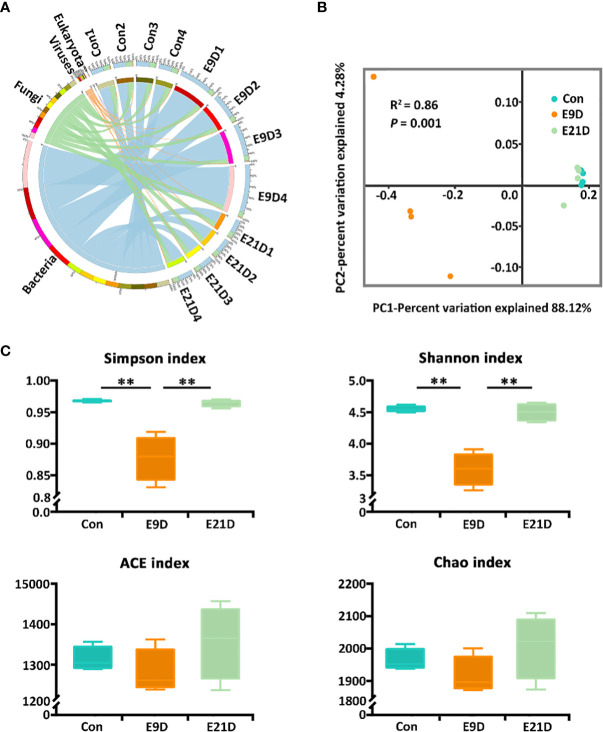
Effects of *Edwardsiella piscicida* infection on kingdom-level composition **(A)**, structure **(B)**, and diversity **(C)** of intestinal microbiota in big-belly seahorses. **(B, C)** represent bacterial intestinal microbiota at the species level. Con represents healthy controls (4.1–4.5 g, PSS); E9D and E21D represent the samples collected on days 9 and 21 of *E. piscicida-* challenged group (4.1–4.5 g, 1 × 10^5^ cfu/mL), respectively (similarly hereinafter). **P < 0.01.

**Figure 2.** Effects of *Edwardsiella piscicida* infection on kingdom-level composition **(A)**, structure **(B)**, and diversity **(C)** of intestinal microbiota in big-belly seahorses. (B, C) represent bacterial intestinal microbiota at the species level. Con represents healthy controls (4.1–4.5 g, PSS); E9D and E21D represent the samples collected on days 9 and 21 of *E. piscicida*- challenged group (4.1–4.5 g, 1 × 10^5^ cfu/mL), respectively (similarly hereinafter). ** *P* < 0.01.

In the published article, there was an error. A repeated and wrong sentence was found in the last paragraph of the **Results** section.

A correction has been made to **3 Results**, *3.6 Molecular pathogenesis of E. piscicida-induced enteritis*. This sentence previously stated: “In addition, seven of the eight KMBs L-Malic acid and L-Glutamate significantly decreased (*P* < 0.05) (Figures 7A; 4D left panel; 5C). In addition, seven of the eight KMBs with central roles were enriched and significantly negatively correlated with two key intestinal microbiota functions, TCS and ABC transporters (*P* < 0.05).”

The corrected sentence appears below:

“In addition, seven of the eight KMBs with central roles were enriched and significantly negatively correlated with two key intestinal microbiota functions, TCS and ABC transporters (*P* < 0.05).”

The authors apologize for these errors and state that they do not change the scientific conclusions of the article in any way. The original article has been updated.

