# Supplementary material for: Edwardsiella piscicida infection reshapes the intestinal microbiome and metabolome of big-belly seahorses: mechanistic insights of synergistic actions of virulence factors
Source: Front Immunol. 2023 May 3;14:1135588. doi: 10.3389/fimmu.2023.1135588 (PMC10193291; doi:10.3389/fimmu.2023.1135588)
Supplement: Supplementary file 1 [file Table_1.docx]

Supplementary Tables

**Table S1** Primers used for real-time quantitative PCR (q-PCR) amplification.

| Gene | Primer sequence | |
| --- | --- | --- |
|  | Sense (5′-3′) | Antisense (5′-3′) |
| *IFN-1* | GGACCTGCGATGCAAACGA | TCTGGAGAAGACGGGATGGC |
| *TNF-α* | TGCTCTGTGCTTAGGCTTGG | CCTTGGCATTGCTGCTGATC |
| *IL-1β* | GAGCTGCGGCGAATCAAT | GGTTGGTGCCCTTGATGTTC |
| *IL-1β receptor* | CACGTCAGGTTGCTTCAAGC | GTCACGCCTACTGTGAGGAC |
| *IL-10* | GTTCCTGAGCACGCAGTTTC | CGGTGTCCAGGTCATCGTTT |
| *IL-2* | ACTCGCTCCACTGGCTTCC | CCCTGCTCTGTGCTCCTCA |
| *Hepcidin* | TCGTGCTCGCCTTTATTTG | GTTGCTTTGCCGCTTGTGT |
| *LEAP* | GCAGGCGGAAGGCAAAGT | TCCACAGCGGGCTCATAC |
| *Piscidin* | CTGGTCACGCTCTTCCTG | TGTCGGTTCTCCCAAGC |
| *LYZ* | TGGTGAATGGCTGGGGAGA | TCCCGCCTTTGAGTTGCTG |
| *TLR-5* | CTCTGCCGCCAAACATTACC | TGTCGCCCAAAACCAAGC |
| *Claudin5* | TTTGGGACTGTCGCTGTG | CACCTGCGGGTTGTAGAAAT |
| *Occludin* | TTGAGGACAAGCCACAGC | CAGGACGGCCAGAAAGTA |
| *ZO-1* | GCTGGATTTGGGTTTGGT | TTCCTCGTCACTGTCCTCTT |
| *18S rRNA* | CCTGAGAAACGGCTACCACATCC | AGCAACTTTAGTATACGCTATTGGAG |

*IL*: *interleukin*; *TNF-α*: *tumor necrosis factor-α*; *IFN*: *interferon*; *LEAP*: *liver-expressed antimicrobial peptide*; *LYZ*: *lysozyme*; *TLR5*: *toll-like receptor 5* (same below).

**Table S2** Detection and scoring system of big-belly seahorses’ intestinal inflammation.

| Group | Features | Description | Score |
| --- | --- | --- | --- |
| External | Increase of height | Normal | 0 |
|  |  | Inhibited | 1 |
|  | Weight | > 5 g | 0 |
|  |  | 4.8-4.9 g | 1 |
|  |  | 4.7-4.8 g | 2 |
|  | Severity of anal inflammation | Normal | 0 |
|  |  | Slight | 1 |
|  |  | Medium | 2 |
|  |  | Serious | 3 |
|  | Feeding status | Can prey on the floating *Mysis* shrimp | 0 |
|  |  | Hunting ability is weak, but can find food | 1 |
|  |  | The ability to hunt is severely reduced, and only food that floats to the mouth is eaten | 2 |
|  |  | Loss of predation | 3 |
|  | Intestinal fluid retention | Normal | 0 |
|  |  | Slight | 1 |
|  |  | Medium | 2 |
|  |  | Serious | 3 |
|  | Respiratory rate | 50-55 | 0 |
|  |  | 45-50 | 1 |
|  |  | 40-45 | 2 |
|  |  | 35-40 | 3 |
| Histological | Number of goblet cells | Normal | 0 |
|  |  | Decrease | 1 |
|  |  | Moderate increase | 2 |
|  |  | Large increase | 3 |
|  | Inflammatory cell count | Normal | 1 |
|  |  | Moderate increase | 2 |
|  |  | Large increase | 3 |
|  | Thickening of lamina propria and muscularis mucosae | Normal | 0 |
|  |  | Slight | 1 |
|  |  | Medium | 2 |
|  |  | Significant | 3 |
| Molecular | *IL-1β* | 0-5 (expression fold, similarly hereinafter) | 1 |
|  |  | 5–10 | 2 |
|  |  | 10–15 | 3 |
|  |  | > 15 | 4 |
|  | *IL-1β receptor* | 0-2 | 1 |
|  |  | 2-4 | 2 |
|  |  | 4-6 | 3 |
|  |  | > 6 | 4 |
|  | *TNF-α* | 0-0.5 | 1 |
|  |  | 0.5-1.0 | 2 |
|  |  | 1.0-1.5 | 3 |
|  |  | >1.5 | 4 |
|  | *IFN-1* | 0-0.5 | 1 |
|  |  | 0.5-1.0 | 2 |
|  |  | 1.0-1.5 | 3 |
|  |  | >1.5 | 4 |
|  | *IL-2* | 0-0.5 | 1 |
|  |  | 0.5-1.0 | 2 |
|  |  | 1.0-1.5 | 3 |
|  |  | >1.5 | 4 |
|  | *IL-10* | 0-0.5 | 1 |
|  |  | 0.5-1.0 | 2 |
|  |  | 1.0-1.5 | 3 |
|  |  | >1.5 | 4 |
|  | *Hepcidin* | 0-5 | 1 |
|  |  | 5-10 | 2 |
|  |  | 10-15 | 3 |
|  |  | > 15 | 4 |
|  | *LEAP* | 0-2 | 1 |
|  |  | 2-4 | 2 |
|  |  | 4-6 | 3 |
|  |  | > 6 | 4 |
|  | *LYZ* | 0-2 | 1 |
|  |  | 2-4 | 2 |
|  |  | 4-6 | 3 |
|  |  | > 6 | 4 |
|  | *Piscidin* | 0-2 | 1 |
|  |  | 2-4 | 2 |
|  |  | 4-6 | 3 |
|  |  | > 6 | 4 |
|  | *TLR5* | 0-0.5 | 1 |
|  |  | 0.5-1.0 | 2 |
|  |  | 1.0-1.5 | 3 |
|  |  | >1.5 | 4 |
|  | *Occludin* | 1.5-2.0 | 1 |
|  |  | 1.0-1.5 | 2 |
|  |  | 0.5-1.0 | 3 |
|  |  | 0-0.5 | 4 |
|  | *Claudin5* | 1.5-2.0 | 1 |
|  |  | 1.0-1.5 | 2 |
|  |  | 0.5-1.0 | 3 |
|  |  | 0-0.5 | 4 |

**Table S3** Statistics of metagenomic data from each sample.

| Sample | Raw data base (bp) | Clean data base (bp) | No host clean data base (bp) | Number of Reads | GC（%） | Q20（%） | Q30（%） | N50（bp） |
| --- | --- | --- | --- | --- | --- | --- | --- | --- |
| Con1 | 11288419584 | 7304333152 | 5541613480 | 19004522 | 43.10 | 96.30 | 91.32 | 1503 |
| Con2 | 12749110882 | 8320390871 | 6368922515 | 21913777 | 43.16 | 96.26 | 91.37 | 1747 |
| Con3 | 14831310994 | 9428266538 | 7378741068 | 25453550 | 43.24 | 96.24 | 91.24 | 1917 |
| Con4 | 13292742912 | 8597712068 | 6776541856 | 23294562 | 43.18 | 96.51 | 91.72 | 1692 |
| E9D1 | 10742114038 | 8498829016 | 6023221780 | 20442501 | 43.28 | 96.26 | 91.21 | 2023 |
| E9D2 | 10134875260 | 8002135746 | 5723109906 | 19480011 | 43.28 | 96.16 | 91.09 | 1965 |
| E9D3 | 11192401592 | 8913383456 | 6444520715 | 21960462 | 43.32 | 96.22 | 91.22 | 2138 |
| E9D4 | 10583957126 | 8269927970 | 5917593764 | 20086587 | 43.23 | 96.41 | 91.52 | 2007 |
| E21D1 | 11172791936 | 7141832835 | 5335853093 | 18248990 | 43.06 | 96.30 | 91.31 | 1519 |
| E21D2 | 13800439912 | 8937949673 | 6867460626 | 23647373 | 43.18 | 96.34 | 91.45 | 1746 |
| E21D3 | 12882712442 | 8465274628 | 6500515936 | 22349130 | 43.13 | 96.15 | 91.07 | 1703 |
| E21D4 | 10285320882 | 7668878313 | 6169563476 | 21398235 | 43.23 | 95.63 | 90.04 | 1434 |

**Table S4** Effects of *Edwarsiella piscicida* infection on various levels of intestinal microorganisms composition.

| sample | Gene number | kingdom | phylum | class | order | family | genus | species |
| --- | --- | --- | --- | --- | --- | --- | --- | --- |
| Con1 | 242189 | 5 | 75 | 101 | 222 | 478 | 961 | 1935 |
| Con2 | 247881 | 5 | 76 | 102 | 215 | 474 | 951 | 1935 |
| Con3 | 254667 | 5 | 78 | 98 | 215 | 477 | 996 | 2013 |
| Con4 | 255017 | 5 | 79 | 100 | 221 | 476 | 955 | 1962 |
| E9D1 | 250358 | 5 | 76 | 102 | 214 | 469 | 943 | 1879 |
| E9D2 | 247513 | 5 | 77 | 102 | 216 | 467 | 928 | 1870 |
| E9D3 | 254443 | 5 | 76 | 101 | 220 | 476 | 954 | 1910 |
| E9D4 | 247493 | 5 | 78 | 106 | 221 | 478 | 975 | 2000 |
| E21D1 | 239005 | 5 | 72 | 99 | 212 | 464 | 938 | 1871 |
| E21D2 | 256229 | 5 | 76 | 102 | 221 | 480 | 972 | 1997 |
| E21D3 | 251463 | 5 | 75 | 99 | 219 | 479 | 995 | 2108 |
| E21D4 | 254425 | 6 | 82 | 106 | 223 | 489 | 998 | 2039 |
| Total |  | 6 | 95 | 119 | 250 | 552 | 1329 | 3171 |
