# Supplementary material for: Edwardsiella piscicida infection reshapes the intestinal microbiome and metabolome of big-belly seahorses: mechanistic insights of synergistic actions of virulence factors
Source: Front Immunol. 2023 May 3;14:1135588. doi: 10.3389/fimmu.2023.1135588 (PMC10193291; doi:10.3389/fimmu.2023.1135588)
Supplement: Supplementary file 3 [file DataSheet_1.docx]

Supplementary Materials and Methods

Raw reads obtained from metagenomic sequencing contain low-quality sequences. In order to ensure the quality of information analysis, Raw Tags need to be filtered using Trimmomatic software to obtain high-quality sequencing data. Then the data were aligned to the third generation of lined seahorse genome by using bowtie2 to remove host information and finally get Clean reads (Table S3) for subsequent information analysis. Metagenomic assembly was performed using the software MEGAHIT (1), contig sequences shorter than 300 bp were filtered, and assembly results were assessed using QUAST (2) software. MetaGeneMark (3) software (<http://exon.gatech.edu/meta_gmhmmp.cgi>, version 3.26) with default parameters was used to identify coding regions in the genome. Redundancy was removed using MMseqs2 (4) software (<https://github.com/soedinglab/mmseqs2>, version 12-113e3). When the similarity threshold was set to 95 % and the coverage threshold was set to 90% annotation, the annotation information of the corresponding sequenced genomic genes was found by BLAST alignment of the protein sequences of the non-redundant genes and the Nr database. The non-redundant genes mentioned above were aligned to sequences in the Nr, KEGG, and VFDB databases to obtain gene annotation information of species, molecular functions and virulence factors.

For extraction of intestinal metabolites, 150 mg tissue of each sample was weighed, added with 1000 μL extracting solution with internal marker (methanol acetonitrile water volume ratio = 2 : 2 : 1, internal marker concentration: 20 mg / L), vortexed for 30 seconds, treated at 45Hz in grinder for 10mins after adding steel balls, and sonicated for 10 min in ice water bath. Then the samples were stationarily incubated  at -20℃ for 1 hour, centrifuged at 12000 rpm for 15min at 4℃, and then 500μL of supernatant was carefully removed into an EP tube and dried in a vacuum concentrator; 160μL of extract solution (acetonitrile to water volume ratio: 1 : 1) were added to dissolve the dried metabolites; after vortex for 30 seconds and sonicate in an ice water bath for 10 minutes, the sample was centrifuged at 4℃, 12000rpm for 15min; 120μL of supernatant was carefully removed into a 2 mL injection vial, and 10μL of each sample was mixed as [quality control](javascript:;) (QC) sample. All samples were loaded for testing according manufacturer's instructions. The raw data collected using MassLynx V4.2 was processed by Progenesis QI software for peak extraction, peak alignment and other data processing operations. Based on the Progenesis QI software online METLIN database, public database and BioMarker's own library for identification, the identification was performed, and the theoretical fragment identification was also performed, the mass number deviation of parent ions within 100 ppm and the mass number deviation of fragment ions within 50 ppm (5). The above steps were performed by BioMarker Biotechnology Co., Ltd. (Qingdao, China).

**References**

1. Li D, Liu CM, Luo R, Sadakane K, Lam TW. MEGAHIT: an ultra-fast single-node solution for large and complex metagenomics assembly via succinct *de* *Bruijn* graph. *Bioinformatics* (2015) 31(10):1674-6. doi: 10.1093/bioinformatics/btv033

2. Gurevich A, Saveliev V, Vyahhi N, Tesler G. QUAST: quality assessment tool for genome assemblies. *Bioinformatics* (2013) 29(8):1072-5. doi: 10.1093/bioinformatics/btt086

3. Zhu WH, Lomsadze A, Borodovsky M. *Ab initio* gene identification in metagenomic sequences. *Nucleic Acids Res* (2010) 38(12):e132. doi: 10.1093/nar/gkq275

4. Steinegger M, Söding J. MMseqs2 enables sensitive protein sequence searching for the analysis of massive data sets. *Nat Biotechnol* (2017) 35(11):1026-8. doi: 10.1038/nbt.3988

5. Wang JL, Zhang T, Shen XT, Liu J, Zhao DL, Sun YW, et al. Serum metabolomics for early diagnosis of esophageal squamous cell carcinoma by UHPLC-QTOF/MS. *Metabolomics* (2016) 12(7):116. doi: 10.1007/s11306-016-1050-5
