# Supplementary material for: Edwardsiella piscicida infection reshapes the intestinal microbiome and metabolome of big-belly seahorses: mechanistic insights of synergistic actions of virulence factors
Source: Front Immunol. 2023 May 3;14:1135588. doi: 10.3389/fimmu.2023.1135588 (PMC10193291; doi:10.3389/fimmu.2023.1135588)
Supplement: Supplementary file 4 [file DataSheet_2.docx]

Supplementary Figures

**
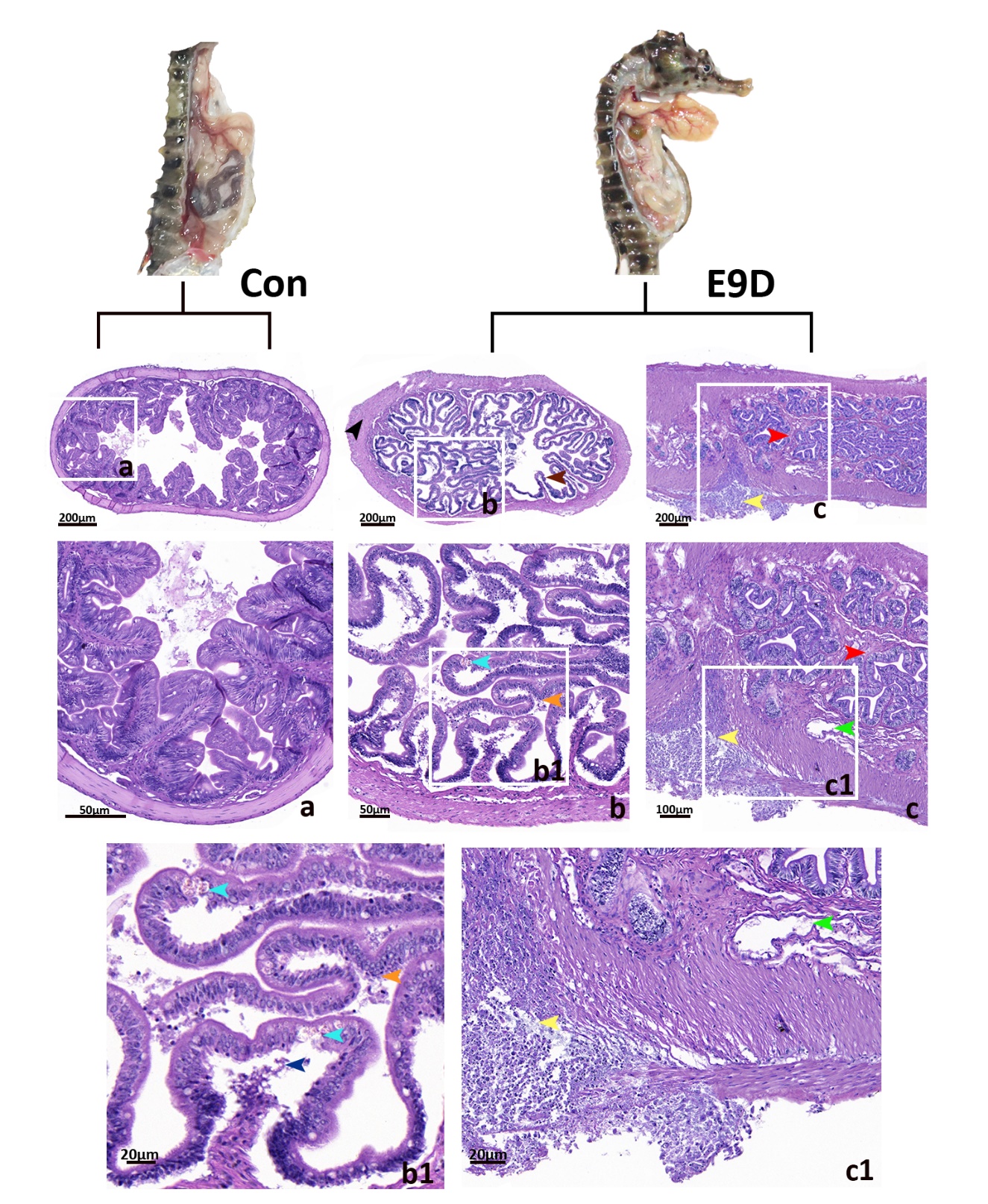
**

**Figure S1** Typical intestinal pathological changes of big-belly seahorse during *Edwardsiella piscicida* infection. (a) Con group; (b, b1, c and c1) samples collected on days 9 of *E. piscicida*-treated group (4.1–4.5 g, 1 × 10^5^ cfu/mL) (E9D). Black arrow: thickened muscularis mucosae; dark red arrow: villus atrophy; blue arrows: focal bleeding; orange arrows: intestinal epithelial dissolution; dark blue arrow: separation between the lamina propria and mucosa; red arrows: thickened lamina propria; yellow arrows: a large amount of inflammatory necrosis in the muscle layer and serosal surface; green arrows: vascular distorted congestion.


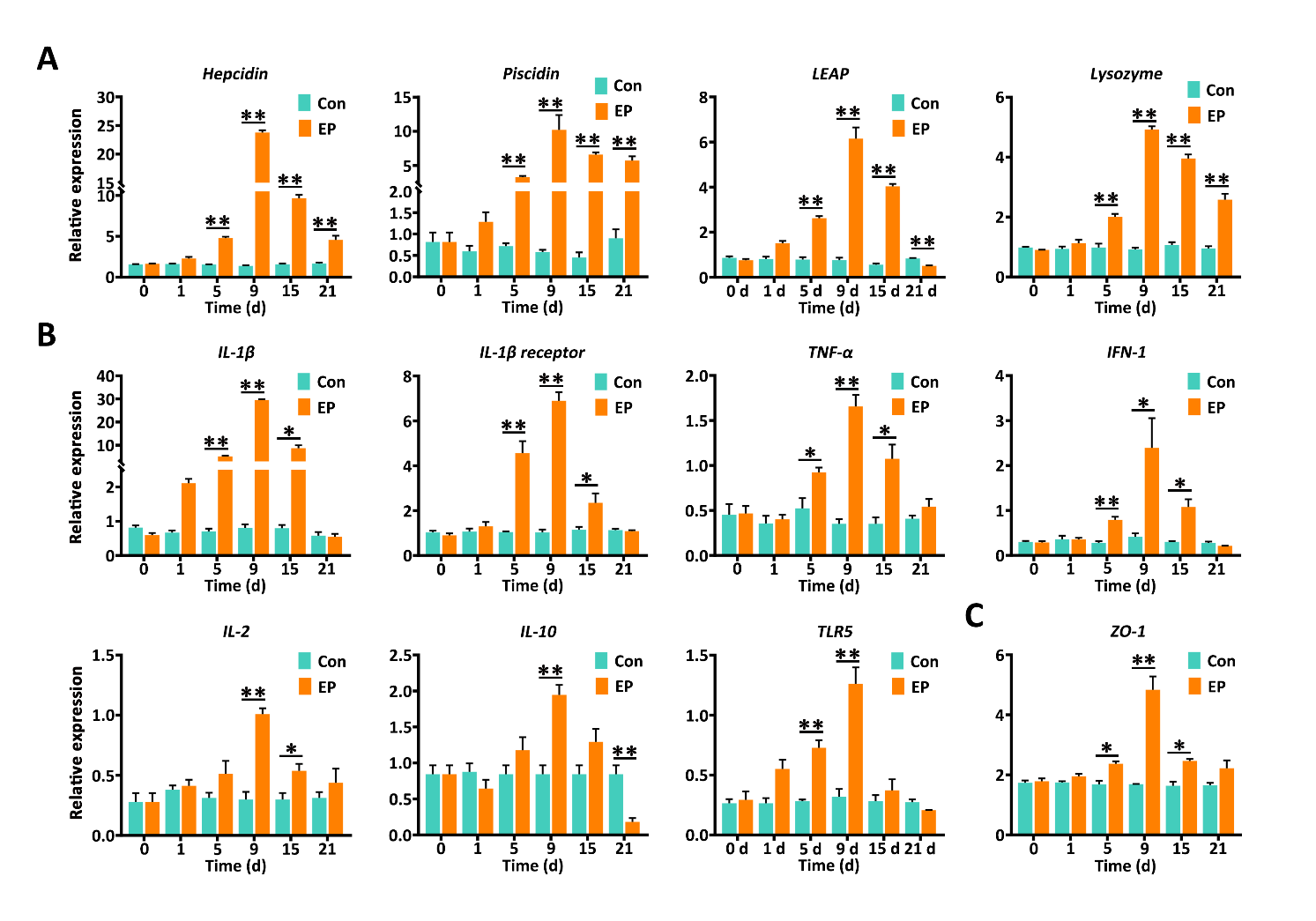


**Figure S2** Effects of *Edwardsiella piscicida* infection on the expression of intestinal antimicrobial peptide gene **(A)**, immune gene **(B)** and tight junction protein gene *ZO-1* (C). * represents *P* < 0.05; ** represents *P* < 0.01 (same below).


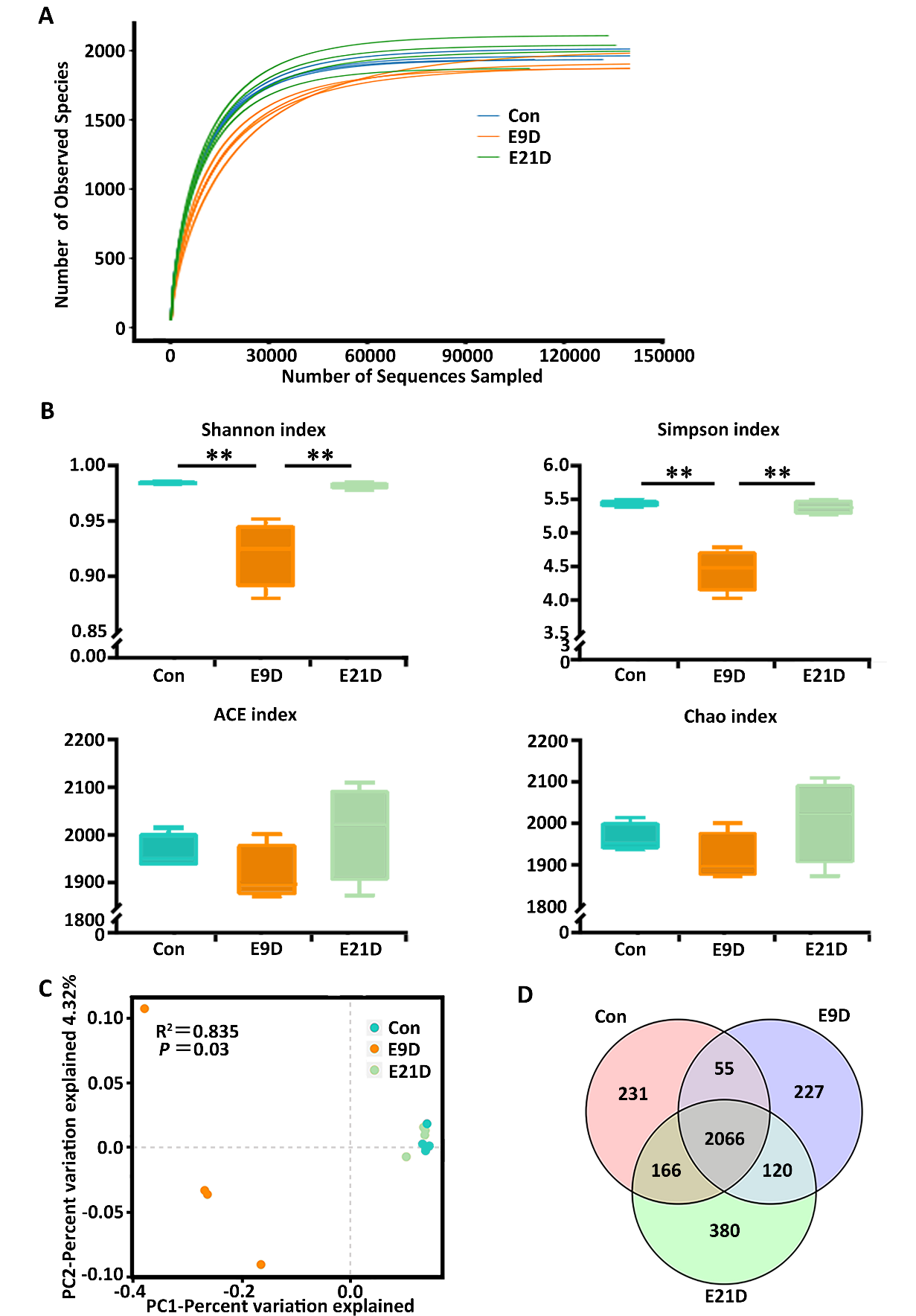


**Figure S3** Effects of *Edwardsiella piscicida* infection on rarefaction curve of intestinal microorganisms species levels **(A),** α-diversity **(B)**, β-diversity **(C)**, and the number of intestinal microbiota composition **(D).**


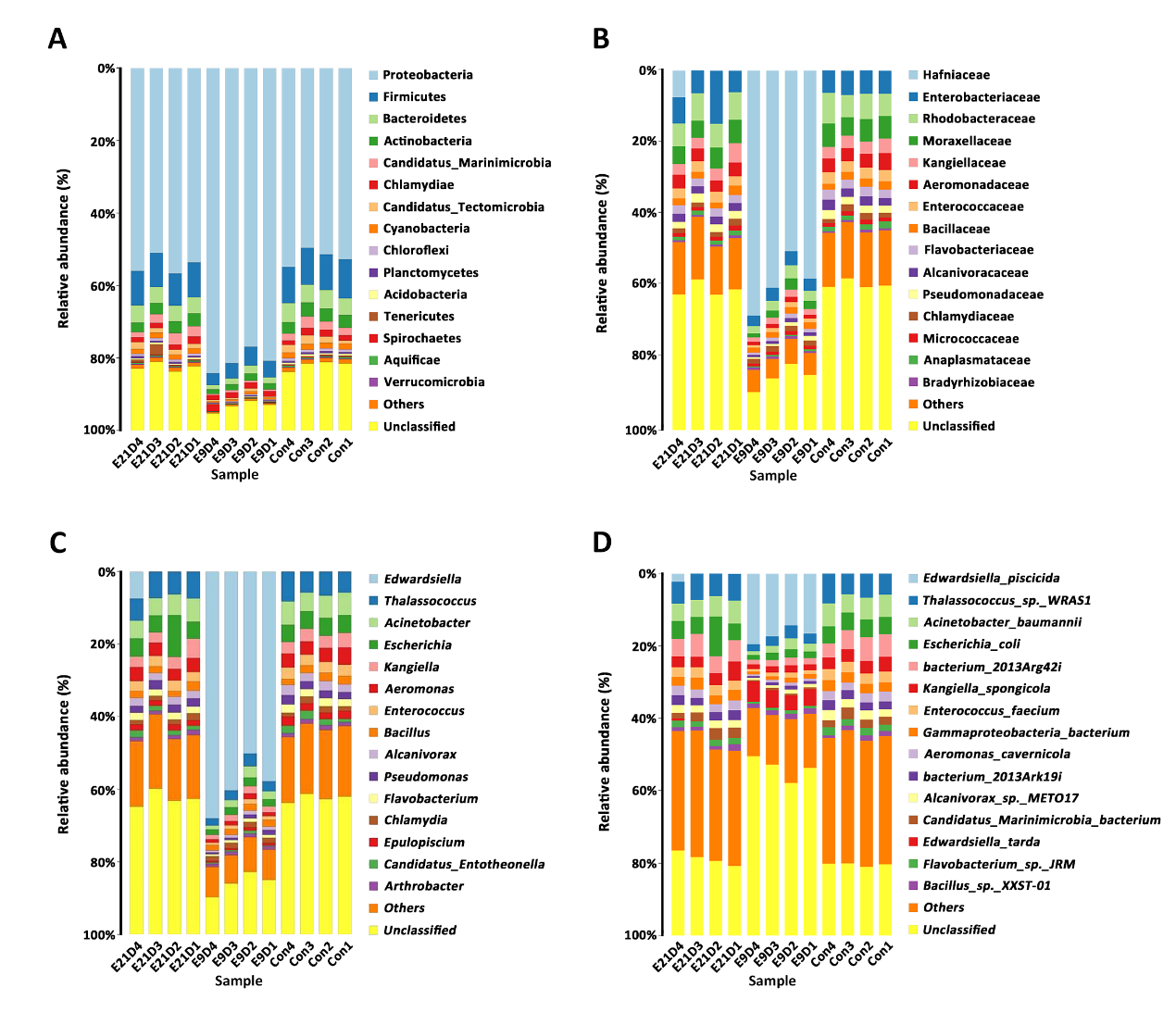


**Figure S4** Effects of *Edwardsiella piscicida* infection on the composition of TOP15 phylum **(A)**, family **(B)**, genus **(C)** and species level **(D)**, the dominant microbiota of intestinal microbiota.


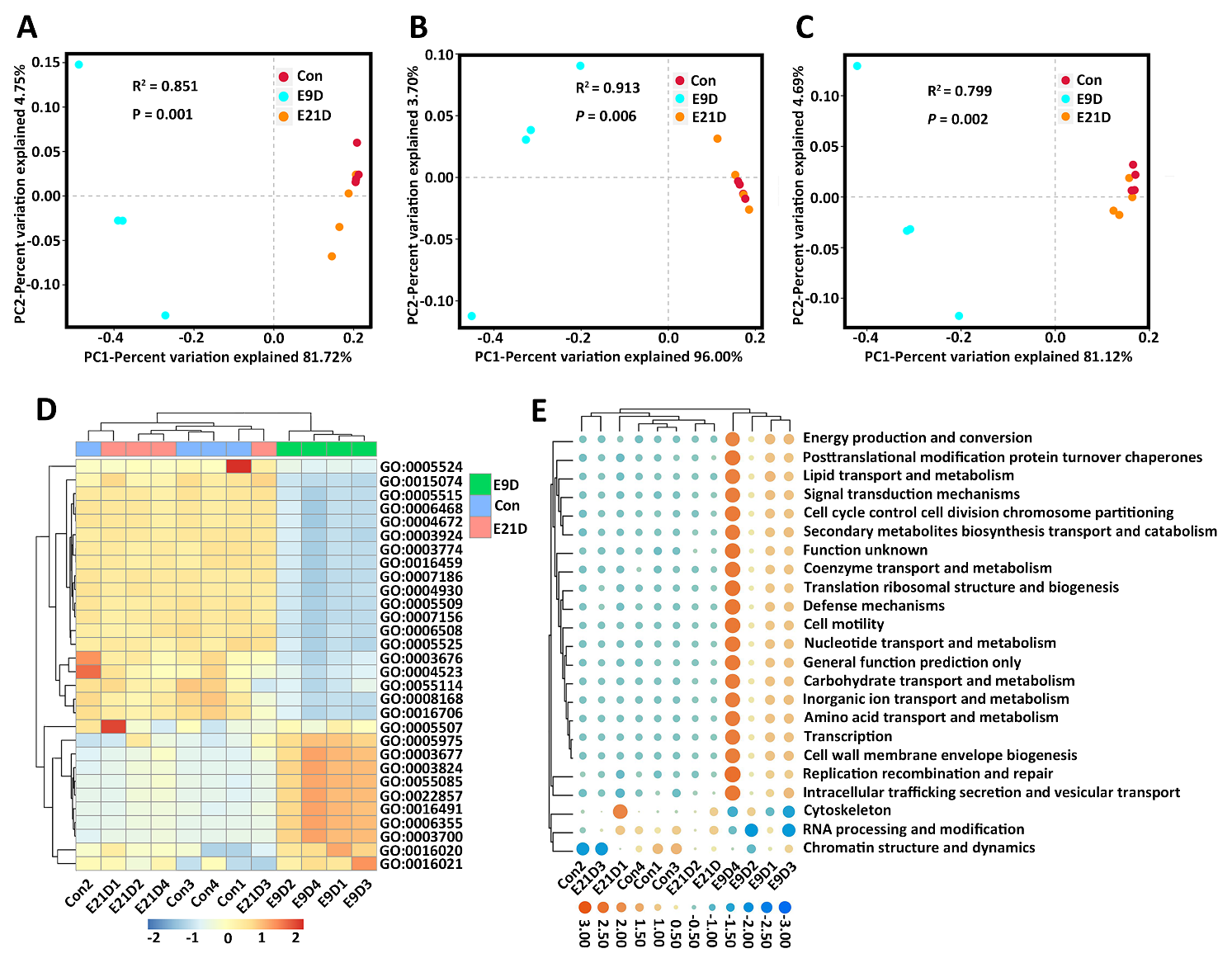


**Figure S5** Effects of *Edwardsiella piscicida* infection on the functional KEGG **(A)**, GO **(B)**, and eggNOG **(C)** structures of the intestinal microbiota and the functional composition of GO **(D)** and eggNOG **(E)**.


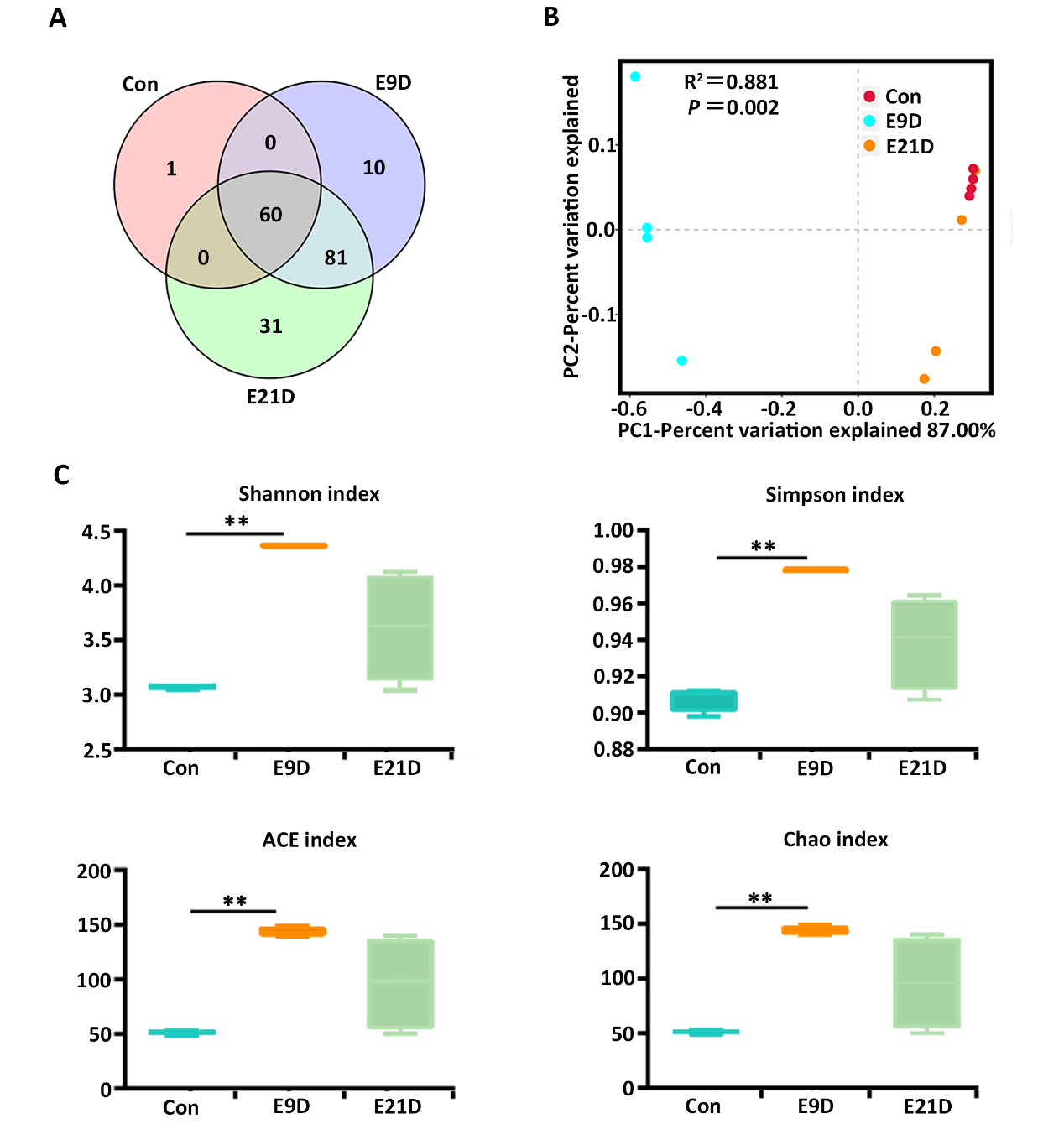


**Figure S6** Effects of *Edwardsiella piscicida* infection on the number of shared and unique virulence factors **(A)** and β-diversity **(B)** and α-diversity **(C)** of the intestinal microbiota.


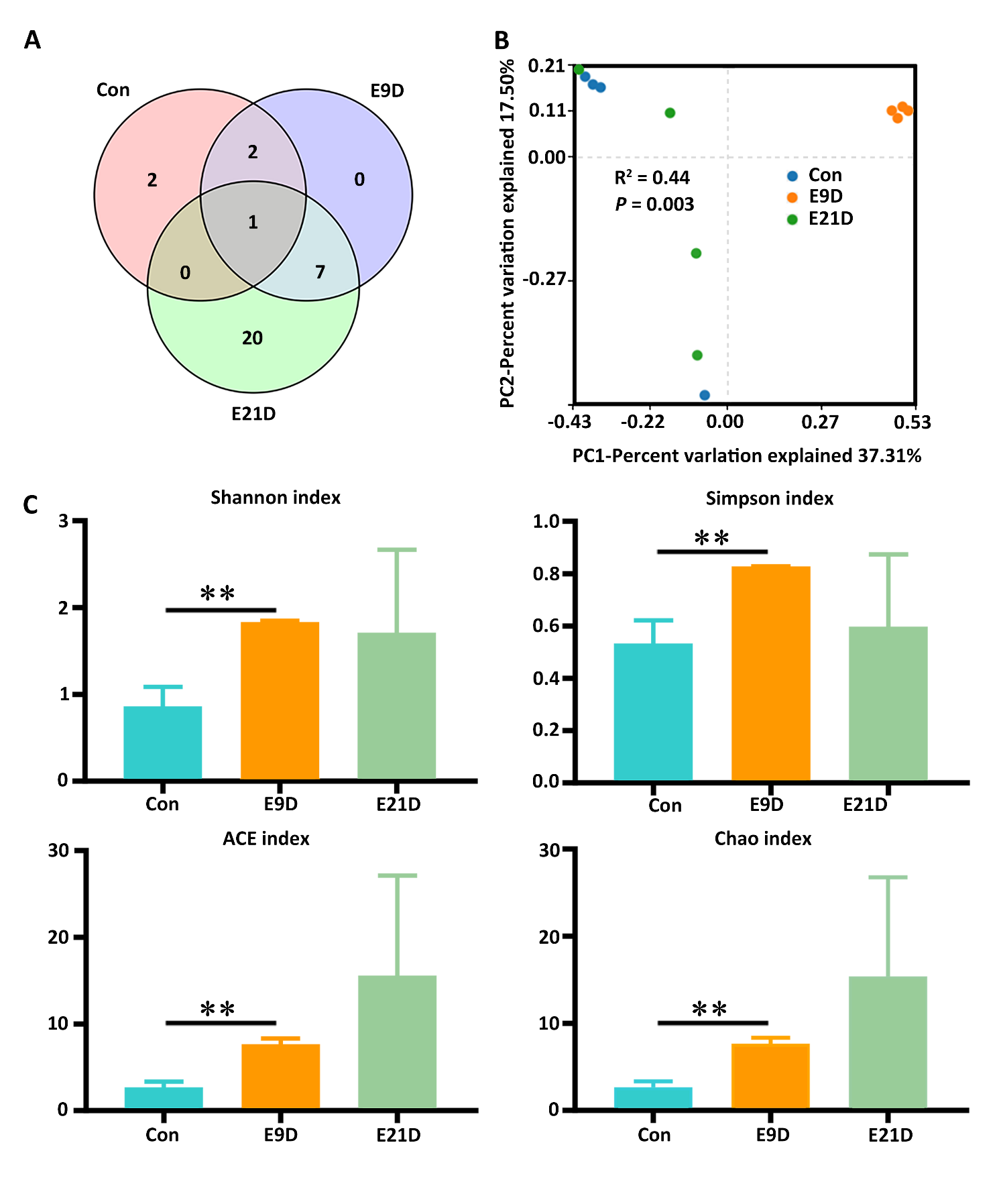


**Figure S7** Effects of *Edwardsiella piscicida* infection on the number **(A)**, β-diversity **(B)** and α-diversity **(C)** of the composition of antibiotic resistance factors in intestinal microbiota.


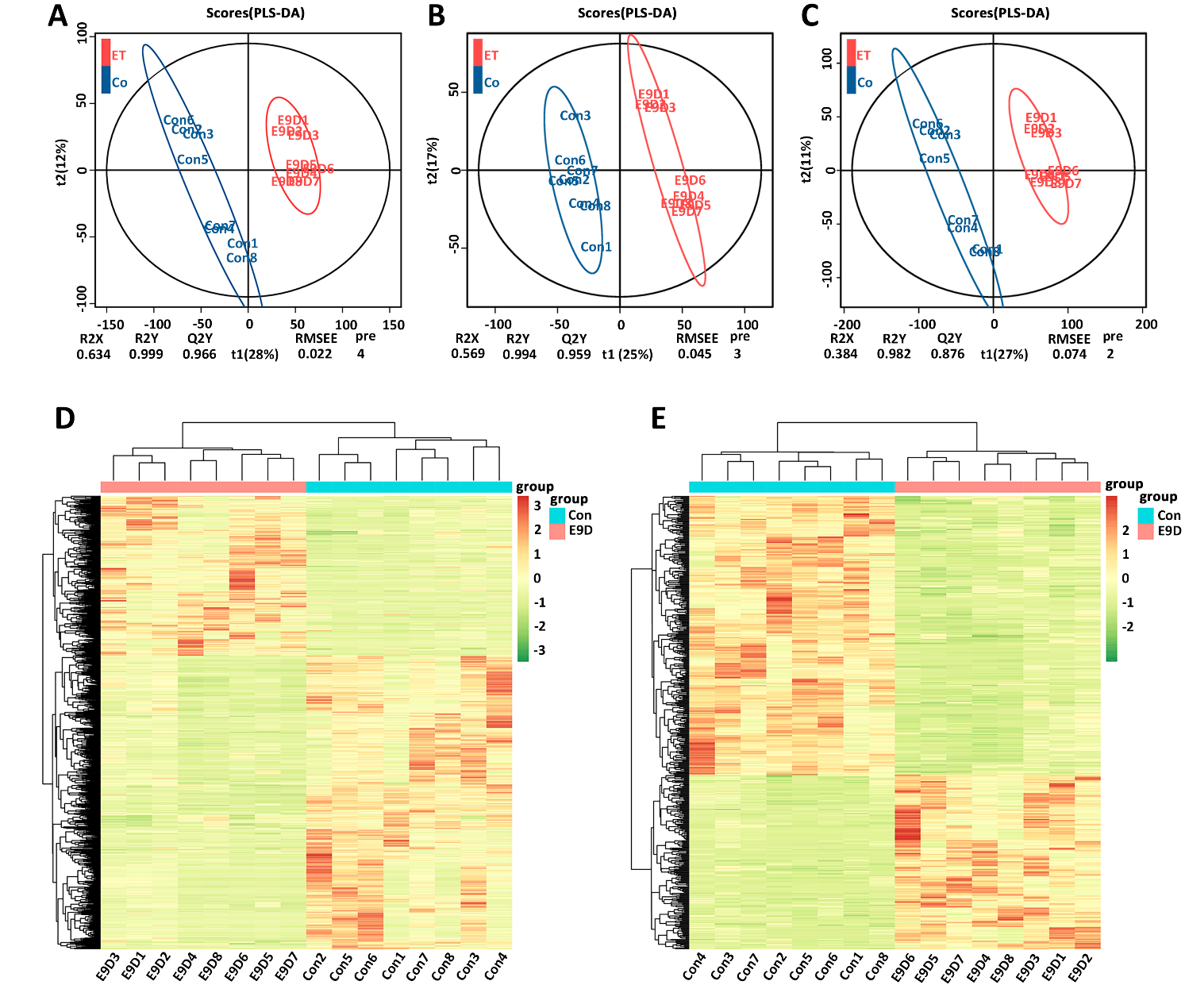


**Figure S8** Effects of *Edwardsiella piscicida* infection on the structure of host positive ion mode (ES+) metabolites **(A)**, negative ion mode (ES-) metabolites **(B)** and total metabolites **(C)**, as well as the compositional abundance of potential metabolic biomarkers (PMBs) (VIP > 1.0, *P* < 0.05) **(D)** and key metabolic biomarkers (KMBs) (VIP > 1.5, *P* < 0.05)**(E)**.
